# Comparison of autoclaving and γ-radiation impact on four spices aroma profiles and microbial load using HS-SPME GC–MS and chemometric tools

**DOI:** 10.1038/s41598-024-56422-6

**Published:** 2024-03-08

**Authors:** Mostafa H. Baky, Nora M. Elkenawy, Heba A. S. El-Nashar, Bishoy Abib, Mohamed A. Farag

**Affiliations:** 1 Department of Pharmacognosy, Faculty of Pharmacy, Egyptian Russian University, Badr CityCairo, 11829 Egypt; 2 Drug Radiation Research Department, National Center of Radiation and Research Technology (NCRRT), Egyptian Atomic Energy Authority (EAEA), Nasr City, 11787 Egypt; 3 Department of Microbiology and Immunology, Faculty of Pharmacy and Drug Technology, Egyptian Chinese University, Cairo, Egypt; 4 Department of Pharmacognosy, Faculty of Pharmacy, Ain Shams University, Abbassia, Cairo Egypt; 5 Chemistry Department, School of Sciences and Engineering, The American University in Cairo, New Cairo, 11835 Egypt; 6 Pharmacognosy Department, College of Pharmacy, Cairo University, Cairo, 11562 Egypt

**Keywords:** Herbal spices, Aroma profile, HS-SPME, Chemometrics, Autoclaving, γ-radiation, Microbial load, Microbiology, Plant sciences, Medicinal chemistry

## Abstract

Herbal spices are widely consumed as food additives owing to their distinct aroma and taste as well as a myriad of economic and health value. The aroma profile of four major spices including bay leaf, black pepper, capsicum, and fennel was tested using HS-SPME/GC–MS and in response to the most widely used spices´ processing methods including autoclaving and γ-radiation at low and high doses. Additionally, the impact of processing on microbial contamination of spices was tested using total aerobic count. GC–MS analysis led to the identification of 22 volatiles in bay leaf, 34 in black pepper, 23 in capsicum, and 24 in fennel. All the identified volatiles belonged to oxides/phenols/ethers, esters, ketones, alcohols, sesquiterpene and monoterpene hydrocarbons. Oxides/phenol/ethers were detected at high levels in all tested spices at *ca*. 44, 28.2, 48.8, 61.1%, in bay leaves, black pepper, capsicum, and fennel, respectively of the total blend and signifying their typical use as spices. Total oxides/phenol/ethers showed an increase in bay leaf upon exposure to γ-radiation from 44 to 47.5%, while monoterpene hydrocarbons were enriched in black pepper upon autoclaving from 11.4 in control to reach 65.9 and 82.6% for high dose and low dose of autoclaving, respectively. Cineole was detected in bay leaf at 17.9% and upon exposure to autoclaving at high dose and γ-radiation (both doses) its level increased by 29–31%. Both autoclaving and γ-radiation distinctly affected aroma profiles in examined spices. Further, volatile variations in response to processing were assessed using multivariate data analysis (MVA) revealing distinct separation between autoclaved and γ-radiated samples compared to control. Both autoclaving at 115 °C for 15 min and radiation at 10 kGy eliminated detected bioburden in all tested spices i.e., reduced the microbial counts below the detection limit (< 10 cfu/g).

## Introduction

From ancient times, spices and herbs have always been recognized as main food ingredients to convey the characteristic flavour of different food products and improve their sensory attributes^1^. Spices are not only used for cooking but traditionally used for health-promoting properties as digestive, carminative, and antimicrobial agents^2^. They widely constitute vital ingredients in food industry such as bakery, confections, dairy, meat, fruit, and vegetable^3^. Since 1990, production of spices has recorded a constant and steady growth of 2.9% per year, achieving 49.6 × 10^6^ tons in 2021 (chilies, peppers, ginger, anise, fennel, coriander, mustard seed, cinnamon, cloves, nutmeg, mace, and cardamoms)^4^.

Owing to their strong preservative and cleansing action, essential oils are considered as raw materials for the pharmaceutical and cosmetic industries represented in creams, shampoos, perfumes, and shower gels^5^. In addition to their economic value, essential oil chemicals exhibit several therapeutic actions against wide range of human disorders such as diabetes, atherosclerosis, heart disease, neurodegenerative diseases, arthritis, and cancer^6^ posing them as nutraceuticals. The essential oil composition in spices is subjected to variation depending on agricultural practices, harvest period, type of processing, drying, and storage, which also could considerably influence their food value and or health effects^7^. Further, spices are susceptible to bacterial contamination and in certain cases spores that are difficult to eliminate affecting their food safety^8^.

Several spices are kept in poor conditions that trigger microbial contamination including mycotoxin-producing moulds, pathogenic microorganisms such as *Salmonella*, and bacterial spores embedded in the soil^4^. Foodborne pathogens such as *Salmonella*, *E. coli*, *Listeria monocytogenes*, *Staphylococcus aureus,* and *Bacillus cereus* species were previously detected in commercial spices^9,10^ likely derived due to improper harvesting, processing, transportation, and storage. Microorganisms such as bacterial spores are important contaminants in the food industry owing to their ability to tolerate processes associated with food industry such as cooking, pasteurization, and disinfection^11^. Some spore-forming bacteria can germinate, and grow, cause food poisoning while other bacteria induce chemical alterations, food degradation and remarkable economic loss^12^. Thus, special attention is needed towards inclusion of spices to ready-to-eat foods which are not exposed to further heat treatments^13^. The fumigation of spices with chemicals such as ethylene oxide, ethylene, dibromide and methyl bromide can produce carcinogenic or mutagenic compounds negatively affecting the human health^14^. Consequently, more safe and proper decontamination technologies are warranted to reduce the bioburden in the case of highly contaminated spices^15^.

For this purpose, autoclaving using steam treatment and irradiation are the most widespread technologies used at a commercial scale; however, in the case of aromatic spices, autoclaving is not the method of choice^16^. Conventional thermal treatment i.e., autoclaving used for food sterilization purposes, though can aid eliminate these pathogens inversely affects food physicochemical properties, mainly color, flavor, and texture^17^. On the other hand, food irradiation by exposure to ionizing radiation such as gamma rays emitted from the radioisotope ^60^Co can be considered as an effective method to eradicate foodborne bacteria without significant changes in food physicochemical and sensory properties^18^. The application of high energy γ-rays as a method of food preservation, decontamination has recently emerged to be safe, effective, and versatile food sectors^19^. These applications cover diverse aspects ranging from inhibition of spice sprouting to extending shelf life of meat, fish, egg, seafood, fruits, vegetables, and dairy products^20^. The international standards and legislations for food γ-irradiation have been approved for more than 60 countries worldwide by organizations such as the World Health Organization (WHO), Food and Agricultural Organization (FAO), and International Atomic Energy Agency (IAEA)^21^. Nevertheless, γ-radiation was found to affect aroma composition as it can induce cell damage^22^ suggestive for changes in spices´ aroma profile in spices, though not broadly addressed in different food matrices in literature.

Typical techniques used for aroma analysis in spices include the use of steam distillation for essential oil isolation that employ heat coupled to analysis using GC–MS, which may cause thermal degradation. In contrast, head space solid-phase microextraction (HS-SPME) coupled with GC–MS provides an efficient analytical tool that enhances volatile components recovery via adsorption on fused silicon fiber with minimal sample preparation and much less amounts^23^. HS-SPME coupled with GC–MS is the most effective method for volatiles profiling in herbal and food products, compared to liquid–liquid extraction and simultaneous distillation–extraction methods^24^. In light of the complexity of datasets generated from different samples following analysis using MS technique, multivariate data analyses implement powerful tools for displaying such large datasets. A variety of multivariate tools are available, including unsupervised principal component analysis (PCA) as well as supervised methods, including orthogonal projection to least squares discriminant analysis (OPLS-DA) designed to develop sample classification and establish metabolite patterns.

Nowadays, the food industry brings about demand for high-quality spices, that is likewise safe to consumers and not much affected by processing methods^7^. From this point of view, the present study aimed to compare the potential effects of autoclaving versus γ-irradiation at different dose levels in major spices of different matrices belonging to seed, leaf, and fruit. Examined spices included bay leaves, capsicum, black pepper, and fennel as analysed using GC–MS and chemometric tools for the first time in literature in such comparative manner.

## Results and discussion

The main goal of this study was to assess γ-irradiation effect compared with autoclaving as sterilization methods to lower bacterial count in major spices with regards to aroma profile. Spices were analysed using GC–MS from 4 major spices (samples codes were listed in Table 1) that included bay leaves, capsicum, black pepper, and fennel treated by autoclave (105 °C for 5 min (AL) and 115 °C for 15 min (AH)), and γ-radiation at low and high doses (5 kGy and 10 kGy, respectively). To aid in samples classification and identify processing impact on volatilome in these spices, chemometric tools were employed including PCA, HCA and OPLS analysis as explained in the next subsections for results in each spice.

**Table 1:** Names and codes of examined spices used in this study.

Name	Code	Autoclaving		Gamma radiation		Control
		Low dose	High dose	Low dose	High dose	
Bay leaf (*Laurus nobilis* L.)	BL	AL	AH	RL	RH	CT
Capsicum fruit (*Capsicum annuum* L.)	CP	AL	AH	RL	RH	CT
Black pepper (*Piper* *nigrum* L.)	BP	AL	AH	RL	RH	CT
Fennel fruit (*Foeniculum vulgare* Mill.)	FL	AL	AH	RL	RH	CT

### GC–MS volatiles profiling from bay leaves post different treatments.

**Figure 1:**
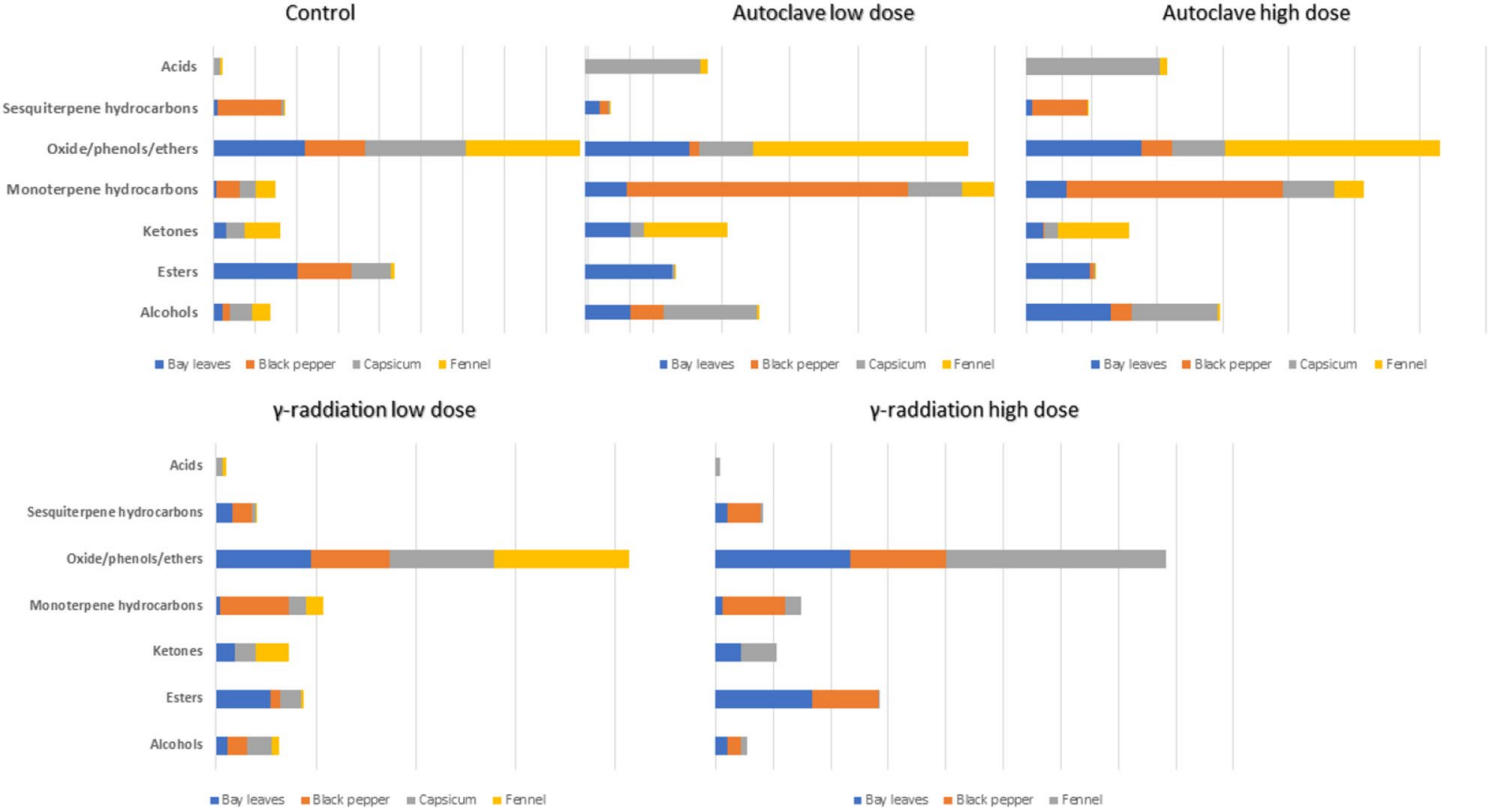
Metabolite classes identified in herbel spices using headspace SPME coupled to GC–MS analysis before and after exposure to autoclaving and γ-raddiation.

GC–MS analysis of tested spices prior to autoclaving and γ-irradiation revealed for 22 major peaks in bay leaf (Table 2, Fig. S1), 34 peaks in black pepper (Table S1, Fig. S2), 23 peaks in capsicum (Table 3, Fig. S3), and 24 peaks in fennel (Table 4, Fig. S4). Identified volatiles belonged to oxides/phenols/ethers, esters, ketones, alcohols, sesquiterpene and monoterpene hydrocarbons (Fig. 1).

**Table 2:** The chemical composition of bay leaf aroma profile recovered from un-treated leaves (BLCT), low-dose autoclave (BLAL), high-dose autoclave (BLAH), low-dose γ-irradiated (BLRL), and high-dose γ-irradiated (BLRH) (n = 3).

Peak No	Average Rt (min)	Average RI	Volatile	Chemical class	BLCT % ± SE	BLAL % ± SE	BLAH % ± SE	BLRL % ± SE	BLRH % ± SE
1	2.65	809	4-Hexen-1-ol	Alcohol	1.00 ± 0.14	8.73 ± 1.67	22.01 ± 0.98	3.95 ± 0.12	2.82 ± 0.47
6	3.78	994	β-Terpineol	Alcohol	0.10 ± 0.01	0.05 ± 0.02	0.03 ± 0.01	0.04 ± 0.001	0.04 ± 0.01
8	4.25	1086	δ-Terpineol	Alcohol	0.86 ± 0.04	1.21 ± 0.02	1.21 ± 0.11	0.50 ± 0.02	0.50 ± 0.01
9	4.29	1094	Terpinen-4-ol	Alcohol	2.44 ± 0.08	2.03 ± 0.09	1.48 ± 0.73	0.75 ± 0.04	0.62 ± 0.28
11	4.38	1112	4-Thujanol	Alcohol	0.13 ± 0.03	0.80 ± 0.24	0.86 ± 0.20	0.04 ± 0.02	0.10 ± 0.001
12	4.52	1143	2,6-Octadien-1-ol, 3,7-dimethyl-, (Z)-	Alcohol	0.09 ± 0.01	0.02 ± 0.01	0.03 ± 0.01	0.01 ± 0.01	0.03 ± 0.01
13	4.60	1158	Nerol	Alcohol	0.02 ± 0.01	0.31 ± 0.08	0.37 ± 0.06	0.10 ± 0.02	0.06 ± 0.01
Total alcohol %					4.64	13.17	25.99	5.39	4.17
15	4.99	1236	Methyl geranate	Ester	38.59 ± 2.31	2.16 ± 0.54	1.47 ± 0.70	22.62 ± 1.30	29.50 ± 0.33
16	5.04	1246	Eugenol acetate	Ester	2.27 ± 0.10	24.40 ± 1.76	17.86 ± 2.63	4.32 ± 0.11	4.19 ± 0.39
Total ester %					40.86	26.56	19.32	26.94	33.69
7	3.82	999	Fenchone	Ketone	6.64 ± 0.78	13.21 ± 0.38	5.41 ± 0.10	9.58 ± 0.22	8.78 ± 0.38
Total ketone %					6.64	13.21	5.41	9.58	8.78
2	3.14	889	α-Pinene	Monoterpene Hydrocarbon	0.08 ± 0.01	1.38 ± 0.67	1.61 ± 0.38	0.21 ± 0.04	0.25 ± 0.03
3	3.21	900	Sabinene	Monoterpene Hydrocarbon	0.86 ± 0.10	9.00 ± 3.01	5.35 ± 1.05	1.35 ± 0.26	1.66 ± 0.16
5	3.68	977	γ-Terpinene	Monoterpene Hydrocarbon	0.53 ± 0.06	1.65 ± 1.04	5.38 ± 2.35	0.27 ± 0.01	0.30 ± 0.01
Total monoterpene hydrocarbon %					1.47	12.03	12.35	1.83	2.22
4	3.53	953	Cineole	Oxide/phenol/ Ether	17.95 ± 1.77	16.90 ± 1.67	29.15 ± 0.75	29.09 ± 0.69	31.32 ± 1.34
10	4.30	1097	Estragole	Oxide/phenol/ Ether	1.76 ± 0.08	2.20 ± 0.13	0.85 ± 0.02	1.77 ± 0.001	1.73 ± 0.06
14	4.73	1185	*E*-Anethole	Oxide/phenol/ Ether	24.38 ± 1.47	11.63 ± 0.50	5.12 ± 0.29	16.99 ± 0.93	13.85 ± 2.00
Total oxide/phenol/Ether %					44.09	30.73	35.12	47.84	46.90
17	5.19	1275	Copaene	Sesquiterpene Hydrocarbon	0.31 ± 0.01	0.67 ± 0.02	0.25 ± 0.04	0.87 ± 0.03	0.60 ± 0.01
18	5.42	1320	α-Longipinene	Sesquiterpene Hydrocarbon	0.08 ± 0.01	0.18 ± 0.05	0.09 ± 0.03	0.30 ± 0.03	0.17 ± 0.02
19	5.43	1322	Aromandendrene	Sesquiterpene Hydrocarbon	1.54 ± 0.01	3.05 ± 0.07	1.19 ± 0.12	6.37 ± 0.24	3.04 ± 0.05
20	5.45	1325	α-Curcumene	Sesquiterpene Hydrocarbon	Traces	0.12 ± 0.05	0.16 ± 0.001	0.04 ± 0.01	0.02 ± 0.01
21	5.75	1384	α-Selinene	Sesquiterpene Hydrocarbon	0.18 ± 0.01	0.24 ± 0.03	0.09 ± 0.04	0.43 ± 0.04	0.22 ± 0.02
22	5.78	1389	Cedrene	Sesquiterpene Hydrocarbon	0.16 ± 0.01	0.05 ± 0.03	0.03 ± 0.001	0.41 ± 0.24	0.18 ± 0.09
Total Sesquiterpene Hydrocarbon %					2.29	4.31	1.81	8.42	4.23

The results were represented in average and SD ( ±).

**Table 3:** The chemical composition of capsicum aroma profile recovered from un-treated fruits (CPCT), low-dose autoclave (CPAL), high-dose autoclave (CPAH), and low-dose γ-irradiated (CPRL) (n = 3).

Peak No	Average RI	Average Rt	Metabolite name	Chemical class	CPCT (% ± SE)	CPAL (% ± SE)	CPAH (% ± SE)	CPRL (% ± SE)
1	665	1.77	Acetic acid	Acid	3.25 ± 0.33	33.97 ± 1.24	40.67 ± 1.37	3.64 ± 0.18
Total acids					3.25	33.97	40.67	3.64
2	702	1.98	2-Propanol, 1-methoxy-	Alcohol	0.15 ± 0.01	1.44 ± 0.18	4.76 ± 1.71	0.10 ± 0.05
3	809	2.66	3-Hexen-1-ol	Alcohol	0.89 ± 0.07	19.31 ± 0.92	15.94 ± 0.54	1.59 ± 0.09
8	951	3.54	β-Terpineol	Alcohol	9.69 ± 0.37	6.79 ± 0.11	5.63 ± 0.17	10.27 ± 0.33
Total alcohol					10.73	27.53	26.32	11.96
7	943	3.40	α-Thujenal	Aldehyde	0.20 ± 0.02	1.66 ± 0.14	0.92 ± 0.05	0.29 ± 0.02
Total aldehyde					0.20	1.66	0.92	0.29
11	1078	4.23	Decane, 2,9-dimethyl-	Aliphatic Hydrocarbon	0.22 ± 0.001	0.54 ± 0.15	0.43 ± 0.17	0.20 ± 0.02
14	1155	4.58	Decane, 2,4-dimethyl-	Aliphatic Hydrocarbon	0.49 ± 0.02	0.05 ± 0.01	0.10 ± 0.04	0.30 ± 0.06
Total aliphatic hydrocarbon					0.71	0.59	0.53	0.50
16	1238	5.00	α-Terpinyl acetate	Ester	18.51 ± 0.52	0.59 ± 0.06	0.49 ± 0.09	10.62 ± 1.78
Total Ester					18.51	0.59	0.49	10.62
10	1000	3.83	Fenchone	Ketone	8.54 ± 0.08	3.02 ± 0.11	3.56 ± 0.22	10.27 ± 0.34
13	1107	4.38	Solanone	Ketone	0.06 ± 0.01	0.75 ± 0.13	0.55 ± 0.04	0.06 ± 0.01
22	1529	6.55	Tumerone	Ketone	0.06 ± 0.02	0.04 ± 0.02	0.01 ± 0.001	0.02 ± 0.02
23	1579	6.75	Curlone	Ketone	0.20 ± 0.03	0.01 ± 0.001	0.02 ± 0.01	0.12 ± 0.02
Total ketone					8.85	3.82	4.14	10.46
4	876	3.05	α-Pinene	Monoterpene Hydrocarbon	0.63 ± 0.02	1.38 ± 0.02	1.32 ± 0.33	0.82 ± 0.06
5	903	3.23	Sabinene	Monoterpene Hydrocarbon	3.48 ± 0.33	5.98 ± 0.68	4.19 ± 0.70	4.18 ± 0.18
6	917	3.29	α-Pinene isomer	Monoterpene Hydrocarbon	3.63 ± 0.12	8.34 ± 0.34	5.03 ± 1.22	3.47 ± 1.70
Total monoterpene hydrocarbon					7.74	15.71	10.55	8.48
9	954	3.55	Cineole	Oxide/Phenol/ Ether	31.15 ± 1.25	13.18 ± 0.38	11.25 ± 0.35	35.88 ± 3.13
12	1098	4.30	Estragole	Oxide/Phenol/ Ether	2.13 ± 0.08	0.40 ± 0.03	0.83 ± 0.15	1.89 ± 0.21
15	1186	4.73	*E*-Anethole	Oxide/Phenol/ Ether	15.57 ± 2.26	2.33 ± 0.21	4.15 ± 0.77	14.44 ± 2.85
Total oxide/ether					48.85	15.91	16.23	52.21
17	1329	5.44	Caryophyllene	Sesquiterpene Hydrocarbon	0.79 ± 0.02	0.08 ± 0.03	0.05 ± 0.01	1.41 ± 0.21
18	1367	5.61	α-Curcumene	Sesquiterpene Hydrocarbon	0.21 ± 0.03	0.07 ± 0.02	0.03 ± 0.01	0.22 ± 0.04
19	1370	5.66	Zingiberene	Sesquiterpene Hydrocarbon	0.04 ± 0.01	0.05 ± 0.02	0.05 ± 0.02	0.05 ± 0.01
20	1381	5.73	l-β-Bisabolene	Sesquiterpene Hydrocarbon	0.04 ± 0.001	0.01 ± 0.001	0.02 ± 0.01	0.06 ± 0.01
21	1407	5.82	Sesquisabinene	Sesquiterpene Hydrocarbon	0.07 ± 0.01	0.02 ± 0.001	0.01 ± 0.001	0.09 ± 0.01
Total sesquiterpene hydrocarbon					1.16	0.23	0.16	1.83

**Table 4:** The chemical composition of fennel aroma profile recovered from un-treated fruits (FLCT), low-dose autoclave (FLAL), high-dose autoclave (FLAH), low-dose γ-irradiated (FLRL), and high-dose γ-irradiated (FLRH) (n = 3).

Peak No	Average Rt (min)	Average RI	Metabolite name	Chemical class	FLCT (% ± SE)	FLAL (% ± SE)	FLAH (% ± SE)	FLRL (% ± SE)	FLRH (% ± SE)
1	1.79	670	Acetic acid	Acid	1.60 ± 0.11	1.99 ± 1.15	2.59 ± 0.31	1.95 ± 0.08	1.53 ± 0.37
Total Acid					1.60	1.99	2.59	1.95	1.53
2	2.6	801	3-Hexen-1-ol	Alcohol	0.07 ± 0.001	0.05 ± 0.03	0.08 ± 0.02	0.06 ± 0.001	0.04 ± 0.02
8	4.35	1106	δ-Terpineol	Alcohol	0.22 ± 0.02	0.02 ± 0.01	0.02 ± 0.01	0.22 ± 0.02	0.20 ± 0.06
13	4.57	1153	Fenchol	Alcohol	0.04 ± 0.03	0.02 ± 0.01	0.06 ± 0.05	0.01 ± 0.001	0.01 ± 0.001
19	5.52	1339	Bergamotol	Alcohol	8.64 ± 0.91	0.40 ± 0.23	0.63 ± 0.11	3.52 ± 1.00	2.18 ± 0.82
			Total Alcohol		8.97	0.49	0.79	3.81	2.43
11	4.47	1132	Linalyl acetate	Ester	1.18 ± 0.19	0.20 ± 0.11	0.20 ± 0.13	0.87 ± 0.01	0.55 ± 0.16
Total Ester					1.18	0.20	0.20	0.87	0.55
6	3.88	1011	Pulegone	Ketone	15.79 ± 0.10	23.79 ± 13.73	21.25 ± 1.28	15.76 ± 0.77	10.98 ± 4.24
7	4.19	1074	Camphor	Ketone	0.39 ± 0.01	0.44 ± 0.26	0.42 ± 0.03	0.35 ± 0.02	0.23 ± 0.09
10	4.45	1128	Solanone	Ketone	Traces	0.01 ± 0.001	Traces	Traces	Traces
23	6.52	1528	Turmerone	Ketone	0.61 ± 0.36	0.02 ± 0.01	0.08 ± 0.02	0.40 ± 0.04	1.06 ± 0.47
24	6.79	1577	Curlone	Ketone	0.02 ± 0.01	0.02 ± 0.01	0.01 ± 0.001	0.02 ± 0.01	0.03 ± 0.001
Total Ketone					16.82	24.27	21.76	16.53	12.30
3	3.08	880	α-Pinene	Monoterpene Hydrocarbon	0.15 ± 0.001	0.25 ± 0.15	0.21 ± 0.02	0.13 ± 0.01	0.10 ± 0.05
4	3.57	958	Limonene	Monoterpene Hydrocarbon	9.17 ± 0.24	9.42 ± 5.44	8.95 ± 0.62	8.24 ± 0.34	5.80 ± 2.72
Total Monoterpene Hydrocarbon					9.31	9.67	9.16	8.37	5.90
5	3.59	962	Cineole	Oxide/ phenol/ether	15.27 ± 0.63	1.73 ± 1.00	1.81 ± 0.16	15.08 ± 0.50	10.71 ± 4.65
9	4.37	1111	Estragole	Oxide/ phenol/ether	9.31 ± 0.25	11.89 ± 6.86	12.45 ± 0.85	7.70 ± 0.68	5.52 ± 1.20
14	4.62	1162	Estragole isomer	Oxide/ phenol/ether	0.03 ± 0.02	0.05 ± 0.03	0.12 ± 0.02	0.80 ± 0.04	0.80 ± 0.24
15	4.65	1168	4-Anisaldehyde	Oxide/ phenol/ether	0.31 ± 0.03	0.27 ± 0.16	0.34 ± 0.05	14.24 ± 13.91	28.56 ± 14.29
16	4.79	1196	*E*-Anethole	Oxide/ phenol/ether	29.84 ± 0.58	48.77 ± 28.16	49.91 ± 2.92	24.86 ± 12.46	26 ± 13.12
12	4.54	1146	2-Allyl-4-methylphenol	Oxide/ phenol/ether	6.34 ± 0.87	0.59 ± 0.34	0.73 ± 0.09	5.21 ± 0.14	5.03 ± 1.54
Total Oxide/phenol/Ether					61.11	63.30	65.36	67.90	76.62
17	5.42	1320	Caryophyllene	Sesquiterpene Hydrocarbon	0.50 ± 0.07	0.03 ± 0.02	0.07 ± 0.03	0.21 ± 0.05	0.34 ± 0.28
18	5.50	1336	*β*-Farnesene	Sesquiterpene Hydrocarbon	0.19 ± 0.09	0.02 ± 0.01	0.02 ± 0.01	0.11 ± 0.05	0.04 ± 0.02
20	5.65	1364	*α*-Bergamotene	Sesquiterpene Hydrocarbon	0.07 ± 0.02	0.01 ± 0.01	0.03 ± 0.01	0.10 ± 0.02	0.06 ± 0.03
21	5.71	1376	*β*-Bisabolene	Sesquiterpene Hydrocarbon	0.24 ± 0.08	0.01 ± 0.01	0.01 ± 0.01	0.15 ± 0.04	0.23 ± 0.08
22	5.86	1404	Cedrene	Sesquiterpene Hydrocarbon	Traces	0.01 ± 0.01	0.01 ± 0.001	0.01 ± 0.001	0.01 ± 0.001
Total Sesquiterpene Hydrocarbon					1.00	0.08	0.15	0.58	0.68

#### Oxides/phenol/ethers

Oxides/phenol/ethers represented by 3 major components anethole, cineol, and estragole were detected at high levels in all tested spices including bay leaves, black pepper, capsicum, and fennel at *ca*. 44.0, 28.2, 48.8, 61.1%, respectively. In bay leaf, significant increase in oxides/phenol/ethers was detected upon exposure to RL and RH to reach *ca*. 47%, compared to significant decline at 30.7 and 35.1% upon exposure to AL and AH, respectively. In fennel, significant increase in total oxide/phenol/ethers was observed from 61.1 to 67.9 and 76.6% upon exposure to RL and RH, respectively. (*E*)-Anethole was detected at high level in bay leaf at 24.3% and to decrease upon exposure to heat and γ-radiation to reach 11.6, 5.1, 16.9, and 13.8% in AL, AH, RL, and RH, respectively, revealing the significant effect of heating, and less observed in case of radiation. (*E*)-Anethole a structural isomer of estragole with distinct sweet, anise-like flavor with potential economic importance as used widely in cosmetics and food industry^25^. (*E*)-Anethole level showed significant reduction from 15.5 to 2.3 and 4.1% upon exposure to AL and AH, respectively in capsicum. Such decline in (*E*)-anethole was though not observed in case of fennel, in which significant increase upon autoclaving from 29.8 to *ca*. 49% using AL and AH was detected, revealing the effect of heat treatment in enhancing fennel aroma and suggestive for a matrix effect in spices. Much lower level of (*E*)-anethole was detected in black pepper at *ca*. 1.1% and slightly enhanced upon exposure to RL and AL to reach 1.9 and 2.6%, respectively. Cineole detected as the chief aroma compound in bay leaf (17.9%) which induce herbal, slightly floral aroma was significantly increased to reach *ca*. 30%, upon exposure to AH, RL, and RH. Such increase in cineole level could be attributed to the fact that γ-radiation induces breakdown of cell wall structure and enhance release of some components^14^. Additionally, cineole level was enhanced in black pepper by autoclaving recording 7.1 and 8.5% upon exposure to AL and AH respectively, compared to 1.5% in control, revealing the effect of autoclaving in enhancing spice aroma. Hence, both autoclaving and γ-radiation can enhance sensory attributes in black pepper and improve its quality, opposite to study which revealed no effect of both sterilization techniques on black pepper aroma^16^. Likewise, RL revealed increase in cineole level in capsicum from 31.1 in control to reach 35.8%, whereas autoclaving showed decline in cineole levels to reach 11.2%. In contrast, cineole as the second major aroma compound in fennel was significantly deceased upon autoclaving and RH from 15.2 to reach 1.8–10.7%, while not being affected by RL. Regarding estragole, both autoclaving and radiation decreased its in capsicum revealing enhanced more safe aroma composition., Estragole, a flavour with susceptible genotoxic effects^25^ was decreased in black pepper from level ca. 25.9% to reach 7.3% upon exposure to RL and almost to trace levels in case of RH suggestive for the potential effect of γ-radiation in improving black pepper aroma safety level. Additionally, estragole level detected in fennel at 9.3% was decreased upon exposure to RL and RH at *ca*. 6–7%, versus an increase up to 12.4% upon autoclaving. Such results conclude that autoclaving can enhance flavour and sensory attributes, albeit can interfere with spice safety, while γ-radiation improved spice safety.

#### Esters

Esters were detected at high levels in bay leaf and black pepper amounting for *ca*. 40.8 and 25.9%, respectively, compared with trace levels in capsicum and fennel. Esters contribute to bay leaf aroma represented by methyl geranate and eugenol acetate detected at 38.5 and 2.2%, respectively. Total esters in bay leaf showed decline upon exposure to autoclaving and γ-radiation as manifested by decline in methyl geranate level upon autoclaving likely due to hydrolysis upon heat treatment^16^. Higher levels of eugenol acetate were detected in bay leaf upon exposure to AL and AH amounted at ca. 24.4 and 17.8%, respectively, compared with control (2.2%) and γ-irradiated leaves (4%). Such increase in eugenol acetate upon exposure to heat treatment is due to increased rate of chemical reactions such as isomerization, and thermal oxidation in edible oils^26^. Such elevation of eugenol acetate upon autoclaving may be due to oxidation and esterification of 2-allyl-4-methyl phenol upon heat treatment which was confirmed by decrease in its level from 6.3% to reach trace level upon autoclaving^27^. Eugenol acetate is a clove-like odoriferous compound with potential antimicrobial, antioxidant, and anti-inflammatory activities^28^, and suggestive that autoclave can increase the antimicrobial action in spices, and should be examined for other spices used as food preservatives for results to be conclusive. α-Terpinyl acetate, a monoterpene ester with sweet, and lavender-like odor is commonly used as flavoring agent and food preservative in food industry owing to its antimicrobial activity^29^. α-Terpinyl acetate represented the major ester in black pepper detected at 25.9% was significantly reduced upon exposure to autoclaving and RL to reach 1.1–5.3%. Likewise, α-terpinyl acetate detected in capsicum at 18.5% showed same pattern to reach trace level upon autoclaving, and to less extent in RL detected at 10.6%. Esters were detected in fennel as linalyl acetate at much lower levels compared to other spices and to likewise decrease upon autoclaving and γ-radiation. From such results, esters appear to decline generally in spices especially upon autoclaving, whereas radiation less affected their levels, except in case of eugenol acetate that showed increase. Hence, γ-irradiation can be used for spice treatment causing slight effect on sensory attributes, while autoclaving improved sensory attributes of leafy spices.

#### Aldehydes/ketone

Ketones were detected at comparable high levels in bay leaf, capsicum, and fennel at 6.6, 8.8, and 16.8%, versus traces in black pepper. Fenchone was the only detected ketone in bay leaf at 6.6% and to increase upon processing using AL, RL, and RH to reach 13.2, 9.5, and 8.7%, respectively. Fenchone, a monoterpene ketone responsible for fennel-like aroma with potential biological activities including anti-inflammatory, antioxidant, wound-healing, antidiarrheal, antifungal, and bronchodilator activities^30^. Likewise, fenchone level in capsicum was enhanced from 8.5 to 10.2% in case of RL. Pulegone was detected as the major ketone in fennel fruit at 15.7% and enhanced upon autoclaving to 23.7 and 21.2% in case of AL and AH, respectively compared to slight decrease with RL and no change with RH. Pulegone, a monoterpene ketone with mint like flavour and possess potential antimicrobial and anti-inflammatory activities^31^. Compared to ketones, aldehydes were detected at trace levels, except in case of capsicum fruit with their level showing no change upon autoclaving or irradiation.

#### Acids/alcohols

Alcohols were detected in the four spices at 3.3, 4.6, 8.9, and 10.7% in black pepper, bay leaf, fennel and capsicum, respectively. Alcohols were represented by 7 peaks in bay leaf showing significant increase in AL and AH at levels 13.1 and 26%, respectively, suggestive that thermal treatment led to an increase in alcohols as observed in esters compared to slight effect observed in case of high and low dose radiation RL (5.4%) and RH (4.1%). 4-Hexen-1-ol (1%) level showed increased to reach 8.7 and 22% with AL and AH, respectively compared to slight increase in γ-irradiated leaves. Such increase in 4-hexen-1-ol, a green leaf alcohol characteristic in green vegetables and their aroma^32^ upon autoclaving as in eugenol acetate and cineole suggestive for enhancement of sensory attributes and leafy spice quality upon heat treatment which has yet to be confirmed using sensory analysis. Likewise, alcohols in black pepper fruit represented by 5 peaks increased from 3.3 to reach 9.8% and 6.2% with AL and AH, respectively and 10.1% and 4.6% in RL and RH, respectively. These results indicate that low dose of autoclaving and radiation can enhance alcohols at higher extent than that observed at larger doses. Linalool was detected at low level (0.7%) in black pepper and enhanced with RL and AL to reach 6.5 and 2.2%, respectively. Likewise, 4-terpineol level showed increased level upon both autoclaving and γ-irradiation. Linalool, a monoterpene alcohol with refreshing, floral and woody scent occurs in aromatic plants and is widely included in cosmetic and food industry and exhibit several biological activities including antimicrobial, anti-inflammatory, anticancer, and antioxidant^33^. Hence, low dose of autoclaving and γ-irradiation are suitable for enhancing the sensory attributes in spices. Likewise, in capsicum, alcohols´ level was enhanced by exposure to high temperature amounting for *ca*. 27% in AL and AH compared to 10.7% in control. 3-Hexen-1-ol level was enhanced from trace level by AL and AH to 19.3 and 15.9%, respectively, and confirming the positive role of autoclaving in improving sensory characters of fruit spices. 3-Hexen-1-ol, an chief leaf compound in green, grass and fresh aroma in vegetable foods specially green tea and its volatility is reported to increase upon exposure to heat^34^. Opposite to the pattern observed in black pepper and capsicum fruits, alcohols showed decrease in fennel fruit from 8.9% to 2.4–3.8 upon irradiation and to reach trace level with autoclaving, and likewise observed in case of bergamotol, a sesquiterpene alcohol in fennel fruit detected at 8.6% versus 2.4–3.8% upon radiation.

Unlike alcohols, acids were detected only in capsicum and fennel represented by acetic acid at 3.2 and 1.6%, respectively. Interestingly, 10- and 20-folds increase in acetic acid upon exposure of capsicum to AL and AH, respectively. Whether heat treatment in capsicum can induce acetogenesis and formation of volatile fatty acids such as acetic acid must be deeply investigated.

#### Mono/and sesquiterpene hydrocarbons

Total monoterpene hydrocarbons were detected in bay leaf, black pepper, capsicum, and fennel at levels reaching 1.4, 11.4, 7.7, and 9.3%, respectively. In bay leaf, monoterpene hydrocarbons level was enhanced by autoclaving to reach comparable level of 123% using AL and AH, respectively. Sabinene (0.8%) and α-pinene (0.1%) levels showed likewise increase upon autoclaving (9.0, 5.3% and 1.3–1.6%, respectively) and γ-irradiated leaves, compared to control, whereas γ-terpinene showed increase in autoclaved leaves only. Sabinene is a monoterpene hydrocarbon with reported antifungal and anti-inflammatory activities^35^. Eleven monoterpene hydrocarbons were detected in black pepper amounting for ca. 11.4% which showed significant increase upon autoclaving to reach 82.6, and 65.9% with AL and AH, and likewise upon irradiation to increase to 34.9 and 21.8% with RL and RH, respectively. Among identified monoterpene hydrocarbons, β-thujene isomer, β-thujene, sabinene, cymene, myrcene, and α-phellandrene levels were enhanced upon autoclaving and γ-irradiation. Similar pattern was observed in capsicum fruit with increase in monoterpene hydrocarbons though to less extent from 7.7% to reach 15.7 and 10.5% upon exposure to AL and AH, respectively especially observed in case of α-pinene and its isomer, a monoterpene hydrocarbon highly distributed in plant essential oil with myriad effects as antimicrobial, anticancer, anti-inflammatory, antioxidant, and gastroprotective^36^. In contrast, monoterpene hydrocarbons were represented in fennel by two peaks identified as α-pinene and limonene.

Similar pattern was observed with sesquiterpene hydrocarbons showing low levels in bay leaf, capsicum, and fennel at 1–2.2%, while detected at much higher levels in black pepper of 30.5%. In bay leaf, sesquiterpene level was enhanced especially upon exposure to AL (4.3%), RL (8.4%) and RH (4.2%). Aromandendrene, a naturally occurring sesquiterpene hydrocarbon belonging to hydroazulene group^37^ that showed increase upon exposure to autoclaving (low dose) and γ-irradiation. Such results were in accordance with the previous work on *Echinodorus macrophyllus* leaf essential oils exposed to γ-radiation which revealed marked increase in sesquiterpene upon exposure to γ-radiation, owing to damage to the leaf cell membranes and liberation of volatile constituents^38^. The level of sesquiterpenes in capsicum was slightly increased in case of RL represented by β-caryophyllene, while level decreased upon autoclaving. Such pattern of increase sesquiteprene levels was though not observed in case of black pepper (30.5%) upon exposure to radiation (16.8 and 2.6 for RL and RH, respectively) and autoclaving (11.6 and 9.5 for AL and AH, respectively) as manifested by decline in β-caryophyllene and α-bisabolene. Caryophyllene is an important component contributing to black pepper sensory aroma^39^. Similar pattern was observed in fennel revealing reduction of sesquiterpenes upon exposure to autoclaving and radiation.

### Multivariate data analyses of spices aroma composition

Owing to generation of huge and complex datasets from MS-based analysis, multivariate data analyses provide powerful tools to better display such large datasets and ease samples classification and identify metabolite markers. The multivariate tools include unsupervised principal component analysis (PCA), in addition to supervised methods, viz., orthogonal projection to least squares discriminant analysis (OPLS-DA) were employed for each spice comparing the impact of autoclaving and γ-irradiation processing on the volatile composition.

#### Unsupervised HCA and PCA Analysis of bay leaf volatiles

**Figure 2:**
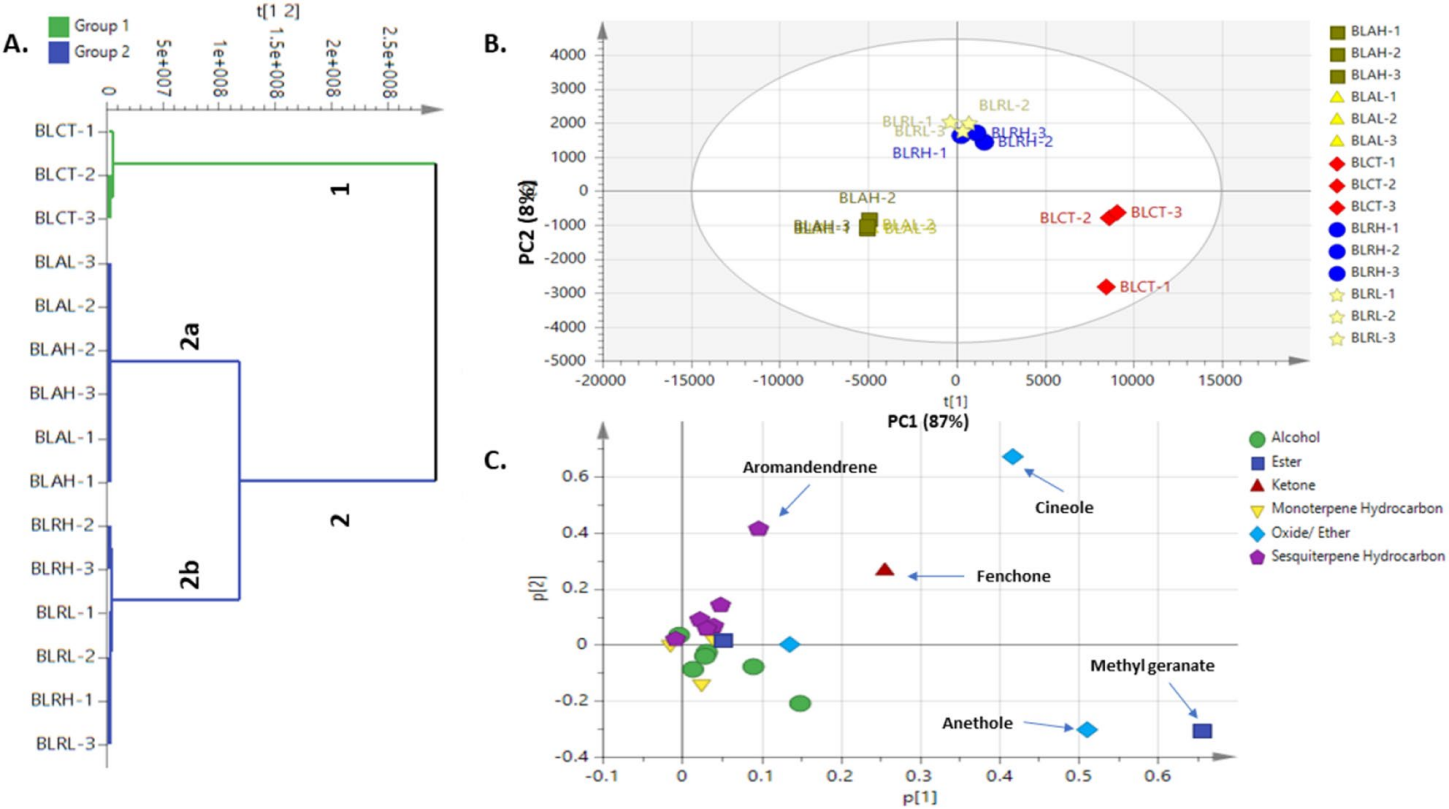
PCA of bay leaf samples' in control and post treatment as described by two vectors of the principal component PC1 (87%) and PC2 (8%). (**A**) HCA dendrogram for different bay leaves. (**B**) Score plot of PC1 vs. PC2. (**C**) Loading plot revealing metabolite variants between clusters (samples codes abbreviations were illustrated in Table 1).

Multivariate data analysis tools were employed to assess processing impact i.e., autoclave, and γ-radiation at low and high doses on aroma profile in an untargeted manner. As illustrated in Fig. 2A, hierarchical cluster analysis (HCA) dendrogram showed two distinct groups in which control samples were clustered together in one group and the processed samples were clustered in two subgroups were autoclaved and γ-radiated samples were intermixed together, suggestive that HCA can clearly distinguish control from processed bay leaves, but not among processing methods i.e., autoclave and γ-radiation. The PCA model accounted for 87% of total variance (Fig. 2B), revealing that bay leaf samples could be differentiated to some extent, being segregated along PC1 accounting for the large variance coverage (95%). Autoclaved leaves were positioned towards the far-left side of PC1, opposite to control samples positioned at far the right side, while γ-radiated leaves were arranged in the middle and suggestive that γ-radiated leaves are closer in aroma composition to that of control leaves. The assessment of the loading plot for PC1 (Fig. 2C) showed the most variant volatile components, mediating for leaves segregation. Methyl geranate and anethole were more enriched in control samples along PC1, and with no markers appearing for autoclaved samples. In contrast, γ-radiated leaves showed higher levels of fenchone and aromandendrene as depicted from their segregation along the upper right side of PC1.

#### Supervised OPLS-DA analysis of bay leaf volatiles

**Figure 3:**
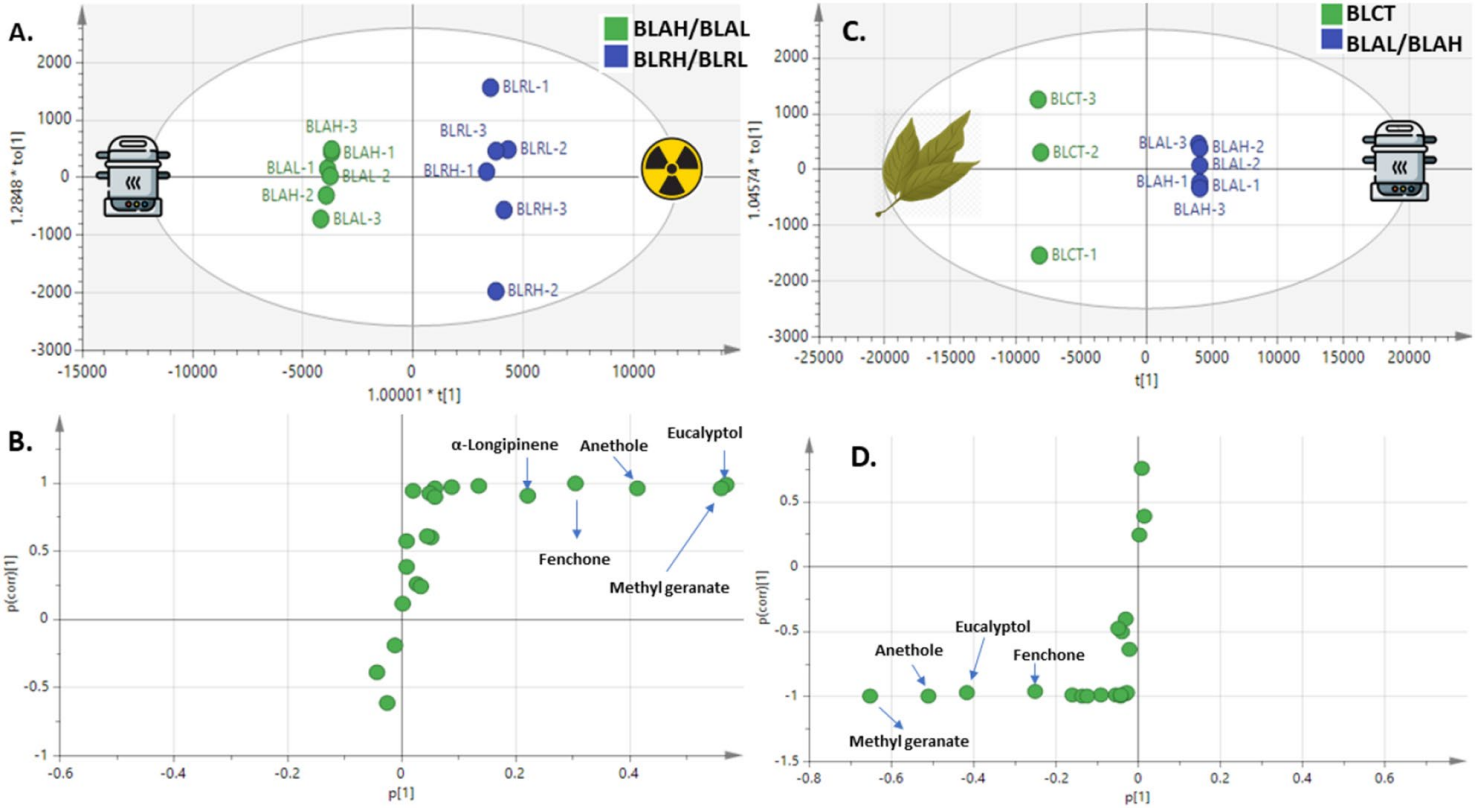
OPLS-DA model for bay leaf irradiated versus autoclaved samples. (**A**) Score plot based on GCMS data. (**B**) S-plot for bay leaves showing covariance p^1^ against the correlation p(cor)^1^ for the variables of discriminating components, (p > 0.05). OPLS-DA model for bay leaf control versus autoclaved samples. (**C**) Score plot based on GCMS data. (**D**) S-plot for bay leaves showing covariance p^1^ against the correlation p(cor)^1^ for the variables of discriminating components, (p > 0.05) (samples codes abbreviations were illustrated in Table 1).

For better assessment of aroma variation among bay leaf volatiles in response to two processing methods, OPLS-DA as a supervised model was employed. OPLS-DA-derived model of autoclaved versus γ-radiated leaves (Fig. 3A) showed Q2 = 0.98 and R2 = 0.99 and p- value of 1.70684e-006, indicating the predictability of the model. OPLS-DA score plot revealed clear segregation of autoclaved samples from radiated samples. The corresponding loading S-plot (Fig. 3B) revealed that E-methyl geranate, E-anethole, fenchone, and cineole were more enriched in γ-radiated samples, and with no markers for autoclaved samples and in agreement with PCA results. Another OPLS-DA-derived modelling of control versus autoclaved samples with Q2 = 0.99 and R2 = 0.99 and p value of 1.42884e-006, indicated strong model predictability with segregation of control sample at the left and autoclaved samples at the right of score plot (Fig. 3C). Inspection of corresponding S-plot (Fig. 3D) revealed that methyl geranate, E-anethole, fenchone and cineole were likewise enriched in control samples, and in agreement with PCA loading plot results (Fig. 2C) and suggestive for stronger aroma in case of control samples.

#### HCA and PCA Analysis of black pepper volatiles

**Figure 4:**
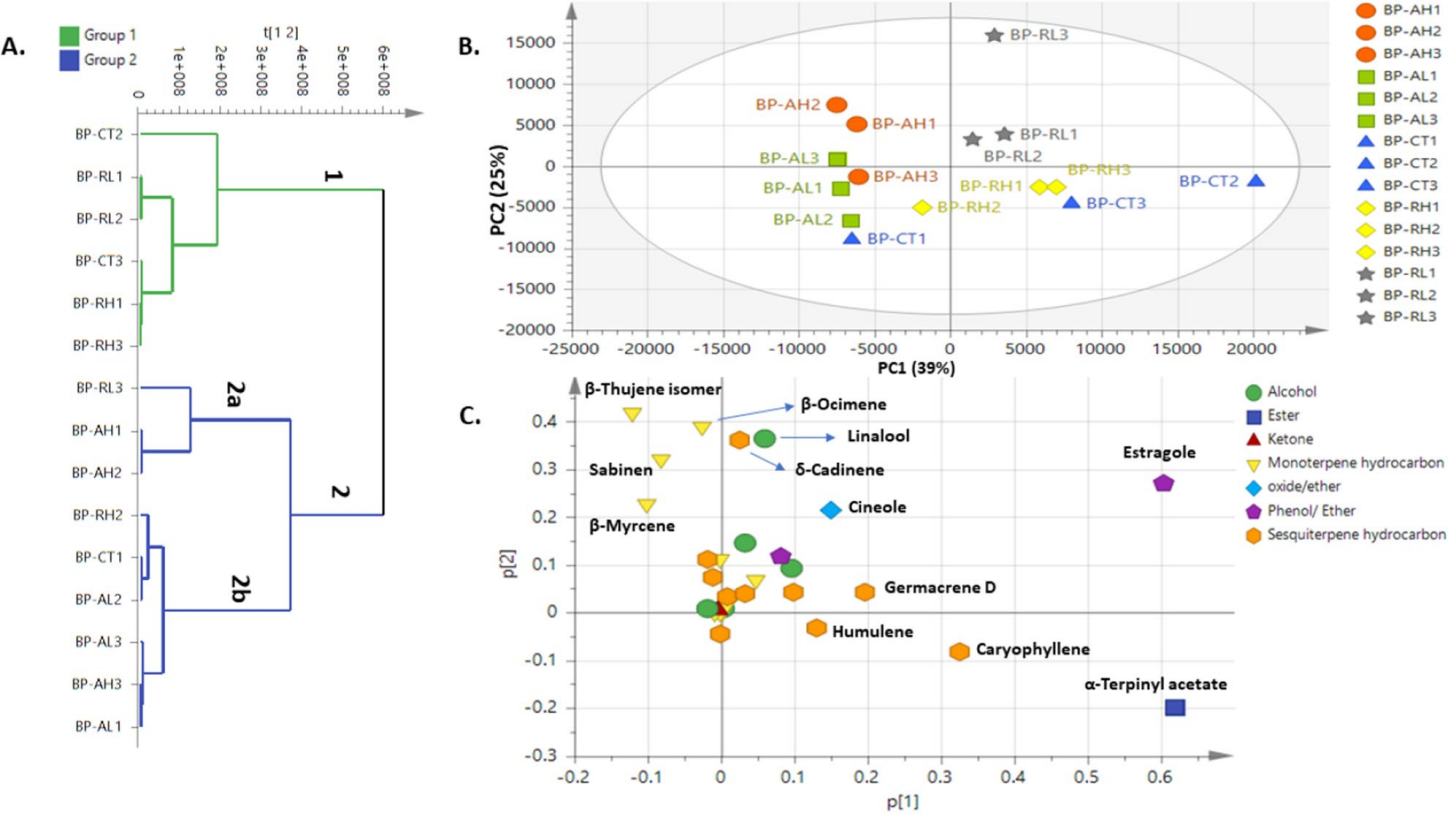
PCA of Black Pepper samples’ clusters as described by two vectors of the principal component PC1 (39) and PC2 (25%). (**A**) HCA dendrogram for different bay leaves. (**B**) Score plot of PC1 vs. PC2. (**C**) Loading plot revealing metabolite variants between clusters (samples codes abbreviations were illustrated in Table 1).

HCA model of black pepper samples (Fig. 4A) showed two major groups though not revealing clear segregation of control samples from treated ones as observed in bay leaf aroma model. Generally, control and γ-irradiated samples were clustered together in group 1, while autoclaved samples with some of control and γ-irradiated were clustered in the other group denoted 2a and 2b. PCA model (Fig. 4B) with two PCs accounted for total variance 64%, showing segregation of autoclaved samples towards the left side of PC1 , whereas radiated samples alongside most control samples were segregated to the right side and in agreement with bay leaf model that radiated samples were closer in aroma composition to that of control seeds though with less variance coverage. The respective score plot (Fig. 4C) revealed enrichment of oxygenated terpenes i.e., estragole, cineole and terpinyl acetate and sesquiterpene hydrocarbons i.e., germacrene D, caryophyllene, humulene in control and radiated samples versus abundance of monoterpene hydrocarbons i.e., β-thujene isomer, sabinene, and β-myrcene in autoclaved samples.

#### OPLS-DA analysis of black pepper volatiles

**Figure 5:**
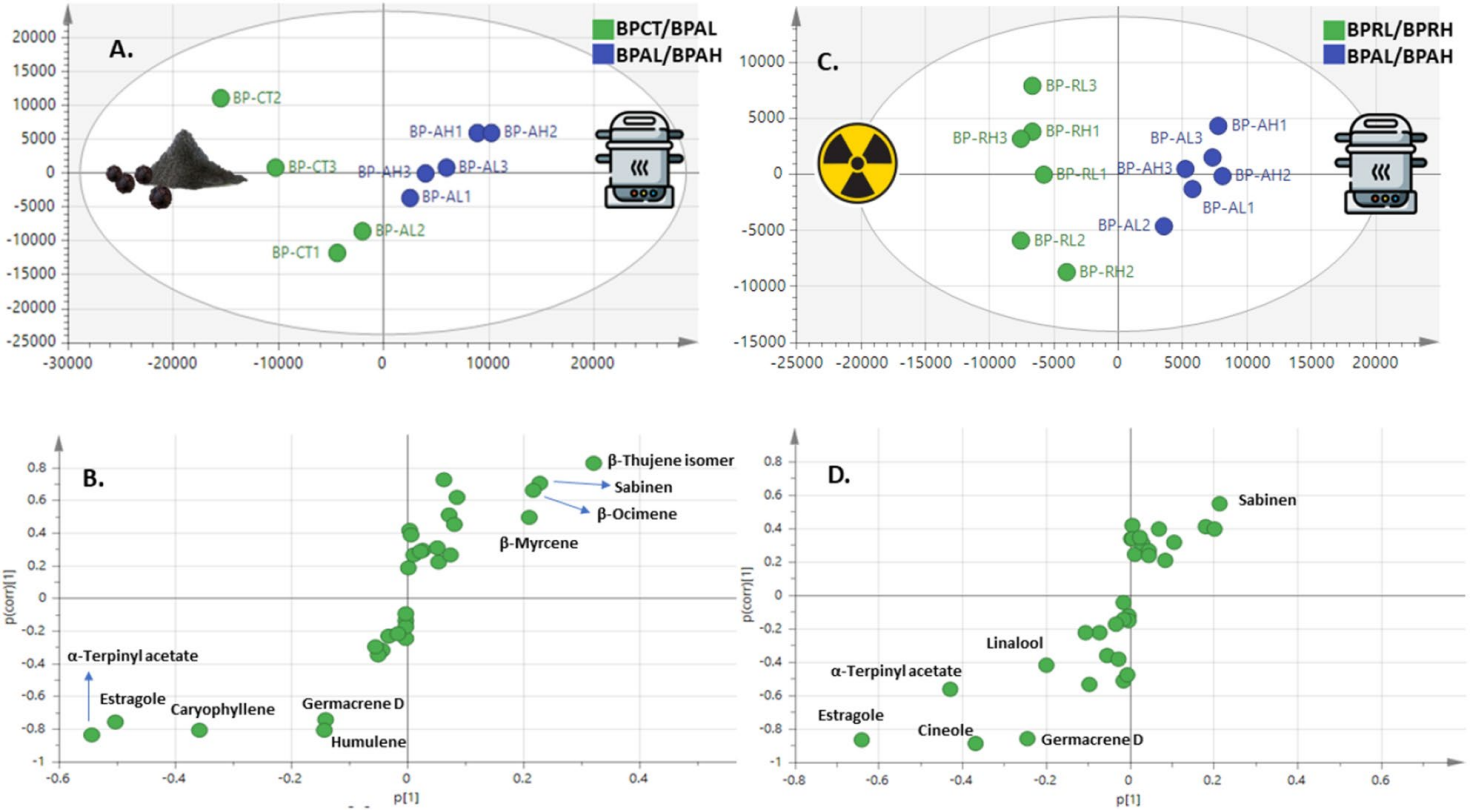
OPLS-DA model for black pepper samples in control and post treatment. (**A**) Score plot based on GCMS data for control versus autoclaved samples. (**B**) S-plot for control and autoclaved black pepper samples showing covariance p^1^ against the correlation p(cor)^1^ for the variables of discriminating components, (p > 0.05). (**C**) Score plot based on GCMS data for autoclaved versus irradiated samples. (**D**) S-plot for black pepper autoclaved and irradiated samples showing covariance p^1^ against the correlation p(cor)^1^ for the variables of discriminating components, (p < 0.05) (samples codes abbreviations were illustrated in Table 1).

Supervised OPLS-AD model was employed to classify between control samples versus autoclaved one at two dose levels (Fig. 5A) revealing segregation of control samples to the left side versus autoclaved segregated at the right side. The OPLS-AD model showed Q2 = 0.62 and 0.61 and R2 = 0.66 and 0.73 and p value less than 0.05. The respective S-plot (Fig. 5B) revealed that monoterpene hydrocarbons i.e., β-thujene isomer, sabinene, and β-myrcene were enriched in autoclaved samples compared to control samples, later more abundant in oxygenated monoterpenes and sesquiterpene hydrocarbons, and in agreement with PCA results (Fig. 4B). Another OPLS-AD model was employed between autoclaved and γ-radiated samples (Fig. 5C) with better differentiation between the two processing methods as indicated by higher Q2 = 0.85 and R2 = 0.95 values, and with p value less than 0.05. The respective S-plot (Fig. 5D) revealed for no markers for autoclaved pepper versus enrichment of oxygenated monoterpenes i.e., linalool, estragole, cineole, and α-terpinyl acetate in radiated samples and suggestive for its improved aroma composition as a processing method for black pepper than autoclaving.

#### HCA and PCA Analysis of capsicum volatiles

HCA model of capsicum specimens (Fig. S5A) revealed clustering of autoclaved samples in one group 1 versus clustering of control samples with radiated samples (low dose) in another group 2. PCA model (Fig. S5B) with two PCs accounted for total variance 100%, revealed segregation of capsicum autoclaved samples at the left side of PC1, while control samples segregated with radiated samples at the right side. The corresponding loading plot (Fig. S5C) revealed that 3-hexen-1-ol was enriched in autoclaved samples and E-anethole, cineole, and α-terpinyl acetate were enriched in both control and γ-irradiated samples.

#### OPLS-DA analysis of capsicum volatiles

Supervised OPLS-AD model was employed between control samples versus autoclaved one (Fig. S6A) for better assessment of changes in aroma composition upon autoclaving, revealing segregation of control samples to the right side while autoclaved were segregated at the left side. The OPLS-AD model showed Q2 = 0.99 and R2 = 0.99 and P value less than 0.05 inferring for the high predictability of the model. The respective S-plot (Fig. S6B) revealed that 4-hexen-1-ol was enriched in autoclaved samples compared to control. Another OPLS-AD model was employed between autoclaved versus control and γ-radiated samples (Fig. S6C) with better differentiation of autoclaved samples. This OPLS-AD model showed Q2 = 0.93 and 0.98 and R2 = 0.95 and 0.99 and P value less than 0.05, indicating a strong model. The respective S-plot (Fig. S6D) revealed that 1-methoxy-2-propanol was enriched in autoclaved samples, whereas cineole, α-terpinyl acetate, and E-anethole were at higher levels in γ-radiated ones.

#### HCA and PCA Analysis of fennel volatiles

HCA model of fennel samples (Fig. S7A) revealed clustering of irradiated samples in one group denoted as 1, versus clustering of control samples with some of the irradiated samples in subgroup 2a, while autoclaved samples were clustered in subgroup 2b. PCA model (Fig. S7B) with two PCs accounting for total variance 82%, revealed segregation of autoclaved samples, control, and some irradiated samples together at the left side of PC1, revealed the failure of the model to differentiated among samples. The corresponding loading plot (Fig. S7C) revealed that (*E*)-anethole was more enriched in autoclaved samples whereas 4-anisaldehyde was enriched in γ-irradiated samples.

#### OPLS-DA analysis of fennel volatiles

For better data assessment, supervised OPLS-AD model was employed between RH samples versus AH ones (Fig. S8A) for better assessment in metabolites variation upon exposure to high doses, revealing segregation of AH samples to the right side, while RH samples were segregated at the left side. The OPLS-AD model showed Q^2^ = 0.61, and R^2^ = 0.99 and P value less than 0.05 revealed the high predictability of the model. The respective S-plot (Fig. S8B) revealed that estragole, pulegone, and (*E*)-anethole were enriched in AH samples, while cineole, 4-anisaldehyde, and 2-allyl-4-methylphenol were abundant in RH samples.

### Total aerobic count in spices post processing

**Figure 6:**
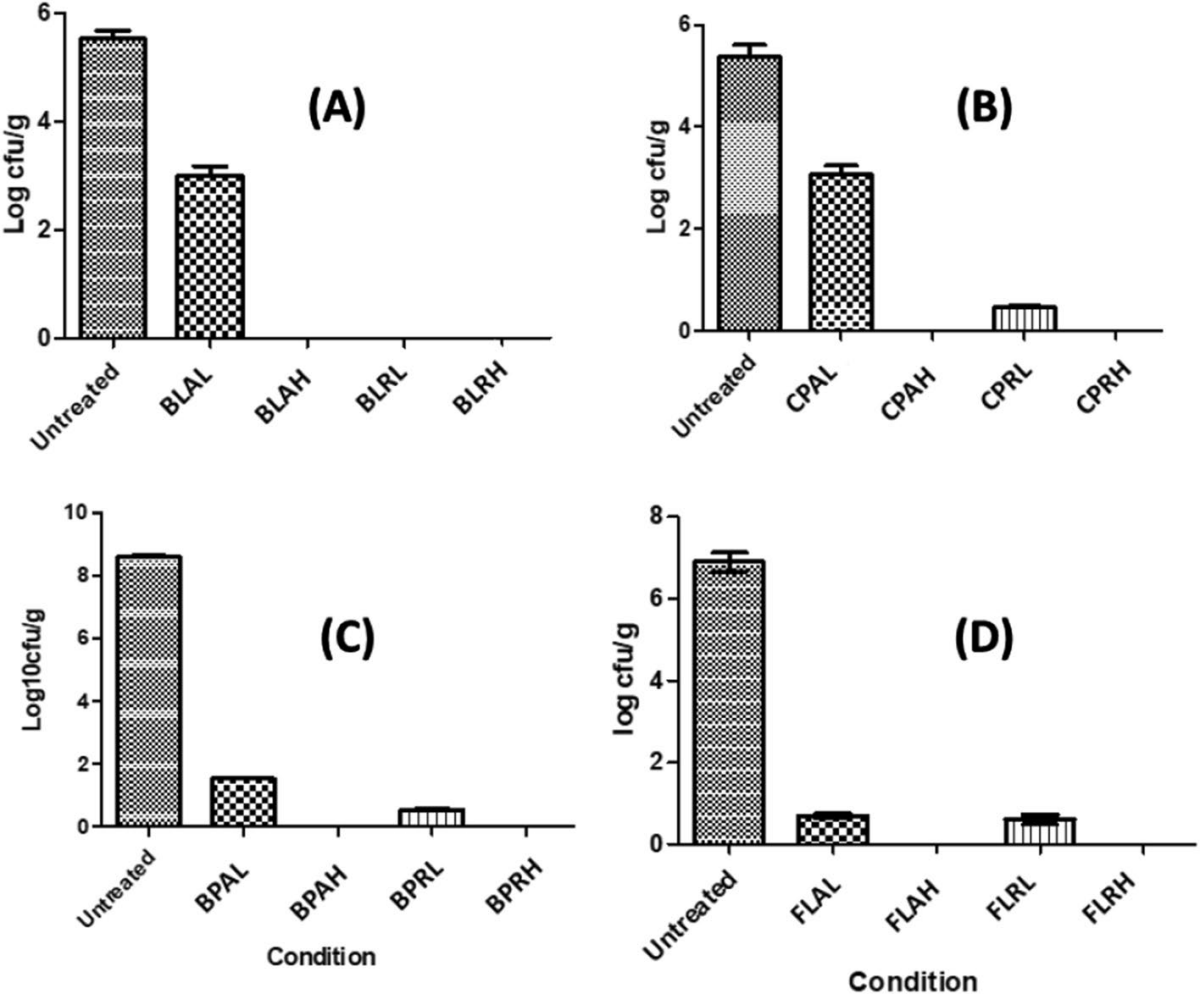
Total aerobic count of tested spices in all tested conditions. (**A**) Bay leaf, (**B**) capsicum, (**C**) black pepper, (**D**) fennel. Significant difference against untreated represented as (#) One-way ANOVA was carried using Graph pad prism followed by Tukeyʾs Multiple Comparisons test (n = 3) results were expressed in average and standard deviation.

To assess the initial bioburden of analysed spices using GC–MS, the total aerobic count was performed, to be compared upon further decontamination processing using either method. Results revealed that black pepper had the highest initial aerobic count (8.3 log cfu/g) among all tested spices, while capsicum had the lowest count (5.3 log cfu/g), as shown in Table 5 and Fig. 6. Limits for aerobic pollutants in spices and herbs are recommended by the International Microbiological Standard to be in the range of 10^1^ to 10^5^ cfu/g using the total aerobic plate count method^40^. After comparing these results to International Microbiological Standards, all spices before treatments were identified to be out of the allowed range with microbiological count that was high above the recommended limits of spices (5 log cfu/g), indicating either poor hygienic standards of preparation, inadequate drying and or inappropriate storage temperature. The difference in microbial profiles between spices can also be attributed to the chemical composition of spices themselves having antimicrobial potential, resulting in a decrease in the intrinsic bioburden^41^.

**Table 5:** Total aerobic count of tested spices (cfu/g).

Spice	Average total aerobic count (cfu/g)				
	Control	AL	AH	RL	RH
Bay Leaf	2 × 10^5^	10^3^	0	0	0
Capsicum	2 × 10^5^	10^3^	0	3	0
Black pepper (BP)	3 × 10^8^	34	0	3	0
Fennel (FL)	9 × 10^6^	5	1	4	0

In our study, two methods of decontamination processing were evaluated to determine their possible effect on the herbal spices’ bioburden. Autoclaving at low temperature (AL) decreased count by (43, 54, 82 and 90%) in case of capsicum, bay leaf, black pepper and fennel respectively Fig. 6. While radiation at low dose (RL) resulted in (91, 92, 93 and 100%) in case of fennel, capsicum, black pepper and bay leaf, respectively Fig. 6. The efficiency of radiation depends mainly on the initial level of contamination and the persistence of the harmful microorganism manifesting a challenge to apply appropriate dose^16^.

Both methods autoclaving at 115 °C for 15 min (AH) and radiation at 10 kGy (RH) eliminated microbial bioburden in all tested spices i.e., reduced the microbial counts below the detection limit (< 10 cfu/g) for all spices with almost 100% reduction of aerobic count compared to untreated samples. Although low irradiation dose was sufficient to reduce microbial load to an acceptable level and eliminate pathogens, radiation dose of 10 kGy resulted in complete decontamination, which was in accordance with^42^ using 10 kGy for complete decontamination of dried chili. An overall dose of 10 kGy is found accepted in many countries worldwide^43^. Both methods applied significantly decreased the microbial count to an acceptable level compared to untreated spices. The effect of γ-radiation and autoclaving on the aroma profile of spices can be pivotal standard in the choice of the decontamination method used, as well as the regimen applied with the one showing best aroma composition to be used.

### Statistical analysis

The results were statistically analyzed for the major metabolites in each spice comparing control to each processing method (Table S2). Values with p < 0.05 are well-thought-out significantly different. Acetic acid was found statistically different between control and autoclaved samples in capsicum. While in bay leaf, fenchone and cineole levels were statistically significant upon irradiation, versus 4-hexen-1-ol upon autoclaving. Moreover, cineole was statistically significant in autoclaved fennel, and sabinene in autoclaved black pepper.

## Conclusion

The impact of two processing methods including autoclaving and γ-radiation on the aroma profile and microbial load of four major spices is reported herein for the first-time using HS-SPME technique. Aroma profiling using HS-SPME/GC–MS analysis was performed before and after exposure to both autoclaving and γ-radiation. Aroma compounds belonging to 6 major chemical classes including oxides/phenols/ethers, esters, ketones, alcohols, sesquiterpene and monoterpene hydrocarbons were identified. 4-Hexen-1-ol, eugenol acetate, cineole, and sabinene were the major aroma belonging to oxygenated and non-oxygenated classes showing increased levels by autoclaving in case of bay leaf. In contrast, monoterpene hydrocarbons viz. β-thujene, sabinene, β-myrcene, and m-cymene levels were significantly increased by autoclaving of black pepper. Autoclaving in case of capsicum appeared to induce acetogenesis as manifested by increase in the level of acetic acid concurrent with increase in 3-hexen-1-ol, and α-pinene levels. Improvement in fennel aroma profile was manifested by increase in (E)-Anethole and pulegone with autoclaving. Regarding changes in aroma profile in context to γ-radiation, several volatiles were enhanced such as cineole, aromandrene in bay leaf, linalool, β-thujene, estragole, and cineol in black pepper, cineole and sabinene in capsicum, and 4-anisaldehyde in fennel. Hence, autoclaving and radiation appeared to exert distinct variations in spices aroma composition, and has yet to be examined regarding differences in health benefits reported for these spices i.e. antimicrobial etc.. On the other side, whether exposure to γ-radiation can negatively affect black pepper safety profile by increasing estragole level has to be further investigated based on these profiling results in other pepper sources. Eugenol acetate, a clove-like odoriferous compound with potential antimicrobial, antioxidant, and anti-inflammatory activities and suggestive that autoclave can increase the antimicrobial action in spices and should be examined for other spices used as food preservatives for results to be conclusive. The effect of γ-radiation and autoclaving on the aroma profile of spices can be pivotal standard in the choice of the decontamination method used, as well as the regimen applied with the one showing best aroma composition to be used. Moreover, applying chemometric analysis revealed a significant variation between autoclaved and radiated samples compared to control which prove the influence of the two processing methods on spices´ aroma profile. Ultimately, both processing methods significantly decreased the microbial count to an accepted level compared to untreated spices.

## Materials and methods

### Plant material

Four major cultivated spices including bay leaves, capsicum, black pepper and fennel spices were provided and identified by Dr. Rupesh K. Deshmukh, Central University of Haryana, India, during October 2022; the spices names and codes are listed in Table 1 and the Latin names follow that listed in the plant list website (http://www.theplantlist.org/). Samples were deposited in tissue culture unit at Phramacognosy Department, Faculty of Pharmacy, Cairo University under codes BL, CA, FL, and BP. The methods in plant collection and experimentation were carried out in accordance with the guidelines prescribed by the American Society of Plant Taxonomists and adopted by the institutional research committee. The dried spices were grinded and kept in the freezer at -20 ºC till preparation for GC–MS analysis. A voucher specimen from the spices was deposited at the College of Pharmacy Herbarium, Cairo University, Cairo, Egypt.

### Processing techniques

#### Autoclaving

Autoclaving of spices was performed by wrapping spices in aluminum foil and placing it inside an Autoclave ALP Model CNBA-75-1-HH, Japan using two regimens: first was at 105 °C for 5 min (AL); whereas the second employed 115 °C for 15 min (AH).

#### Gamma radiation

Spices were irradiated at doses (5 kGy and 10 kGy) in ^60^Co-source Indian gamma cell (GE 4000A) located at the National Center of Radiation and Research Technology (NCRRT), Egyptian Atomic Energy Authority (EAEA), Egypt. The dose rate was 1kGy/h at the time of experiment.

### SPME and chemicals

Special fibers used in extraction of volatiles by SPME are stable flex coated with divinylbenzene/carboxen/polydimethylsiloxane (DVB/CAR/PDMS, 50 µm/30 µm, stable flex 24 Ga, fiber L 1 cm) or PDMS (polydimethylsiloxane) were purchased from Supelco (Oakville, ON, Canada). All volatile standards were purchased from Sigma-Aldrich (St. Louis, MO, USA)^44,45^.

### SPME–GC–MS volatiles analysis

Freeze dried finely powdered spices (100 mg) were placed in SPME screw-cap vials (1.5 mL) spiked with 10 µg (*Z*)-3-hexenyl acetate with fibers inserted manually above and placed in an oven kept at 50 °C for 30 min^46–48^. The spiked aroma compound Z- 3 Hexenyl acetate 10 ug/ml was not injected on the column but prior to volatiles collection step using SPME as volatiles collection is done manually by inserting the fiber above the spice powder. HS-SPME analysis of the volatile compounds was performed as reported in^49^ with slight modifications. The fiber was subsequently withdrawn into the needle and then injected manually into the injection port of a gas chromatography–mass spectrometer (GC–MS). GC–MS analysis was adopted on GC Shimadzu with a DB-5 column (30 m × 0.25 mm i.d. × 0.25 µm film thickness; Supelco) and coupled to a quadrupole mass spectrometer. The interface and the injector temperatures were both set at 220 °C. Volatile elution was carried out using the following gradient temperature program: oven was set at 40 °C for 3 min, then increased to 180 °C at a rate of 12 °C/min, kept at 180 °C for 5 min, finally increased at a rate of 40 °C/min to 240 °C and kept at this temperature for 5 min. Helium was utilized as a carrier gas with a total flow rate of 0.9 mL/min. For ensuring complete elution of volatiles, SPME fiber was prepared for the next analysis by placing it in the injection port at 220 °C for 2 min. Three different samples for each accession were analyzed under the same conditions to assess biological replicates, and blank runs were made during sample analyses. The mass spectrometer was adjusted to EI mode at 70 eV with a scan range set at m/z 40–500.

### Volatiles identification and multivariate data analyses

Identification of volatile components was performed by comparing their retention indices (RI) in relation to n-alkanes (C6-C20), mass matching to NIST 11.0, WILEY library database 9.0 and with standards if available. GC–MS files were converted to. netcdf file format using through MS Convert option in Shimadzu program, then to abf files utilizing ABF converter (https://www.reifycs.com/AbfConverter/). In that regard, data analysis was performed using MS dial software (http://prime.psc.riken.jp/compms/msdial/main.html) according to the following parameters: mass range (0–220 Da), MS1 tolerance for alignment (0.015 Da), retention time (0–18 min), minimum peak height (1000), sigma (0.7), accurate mass tolerance (MS) 0.01 Da, and peak area1000. Peak abundance was exported for multivariate data analysis where final ID and metabolites were Pareto scaled using SIMCA 14.1 (Umetrics, Umea, Sweden) in which the obtained data were subjected to principal component analysis (PCA) and orthogonal partial least squares discriminant analysis (OPLS-DA). PCA was carried out to show the variance of metabolites amongst different samples whilst information on differences in the metabolite composition can be professed by OPLS-DA plot^48^. Peaks were first deconvoluted using AMDIS software (www.amdis.net)^50^ before mass spectral matching. Peak abundance data were exported for multivariate data analysis by extraction using MS-Dial software under same conditions cited in^51^. Markers were subsequently identified by analyzing the S-plot, which was declared with covariance (p) and correlation (pcor). All variables were mean-centered and scaled to Pareto variance. Model validation was assessed by computing the diagnostic indices, viz. Q2 and R2 values, p value and permutation testing.

### Total aerobic plate count

The total aerobic count of 4 samples was performed using^52^ method. A stock solution of the sample was prepared by weighing 1 g of the sample into 9 ml of 0.1% sterile peptone water and shaken thoroughly. A ten-fold serial dilution of the sample was made. This was done until 10^−8^ dilution was achieved, 0.1 ml was then pipetted from the 10^−8^ dilution onto the surface of each of two Petri dishes containing 20 ml of a solidified and sterile Plate Count Agar (PCA), and then spread evenly with sterile glass spreader. The plates were then incubated for a maximum of 48 h at 37 °C (including the control plates). The counting of the colonies was done and expressed as cfu/g. Experiment was done in triplicates.

### Statistics

Data were expressed as mean ± standard error (SE). GraphPad Prism® software program was used for analysis, one-way analysis of variance (ANOVA) test followed by Tukey- Kramer multiple comparison’s test was selected to carry out all statistical tests.

### Supplementary Information

Supplementary Information.

## Data Availability

All data generated or analyzed during this study are included in this published article [and its supplementary information files].
