# Supplementary material for: Comparison of autoclaving and γ-radiation impact on four spices aroma profiles and microbial load using HS-SPME GC–MS and chemometric tools
Source: Sci Rep. 2024 Mar 8;14:5752. doi: 10.1038/s41598-024-56422-6 (PMC10923872; doi:10.1038/s41598-024-56422-6)
Supplement: Supplementary file 1 — Supplementary Information. [file 41598_2024_56422_MOESM1_ESM.docx]

**Supplementary materials**

**Comparison of autoclaving and γ-radiation impact on four spices aroma profiles and microbial load using HS-SPME GC-MS and chemometric tools**

**Mostafa H. Baky^1^, Nora M. Elkenawy^2,3^, Heba A.S. El-Nashar^4^, Bishoy Abib^5^, Mohamed A. Farag ^6^***

*^1^ Department of Pharmacognosy, Faculty of pharmacy, Egyptian Russian University, Badr city, 11829, Cairo, Egypt.*

*^2^Drug Radiation Research Department, National Center of Radiation and Research Technology (NCRRT) Egyptian Atomic Energy Authority (EAEA) Cairo, 11787, Egypt*

*^3^Department of Microbiology and Immunology, Faculty of Pharmacy and Drug Technology, Egyptian Chinese University*

*^4^Department of Pharmacognosy, Faculty of Pharmacy, Ain Shams University, Abbassia, Cairo, Egypt*

*^5^Chemistry Department, School of Sciences and Engineering, The American University in Cairo, New Cairo 11835, Egypt.*

*^6^Pharmacognosy Department, College of Pharmacy, Cairo University, 11562 Cairo, Egypt.*

*Corresponding author at: Cairo University, College of Pharmacy, Department of Pharmacognosy, Egypt.
E-mail addresses: [mohamed.farag@pharma.cu.edu.eg](mailto:mohamed.farag@pharma.cu.edu.eg), (M.A. Farag).


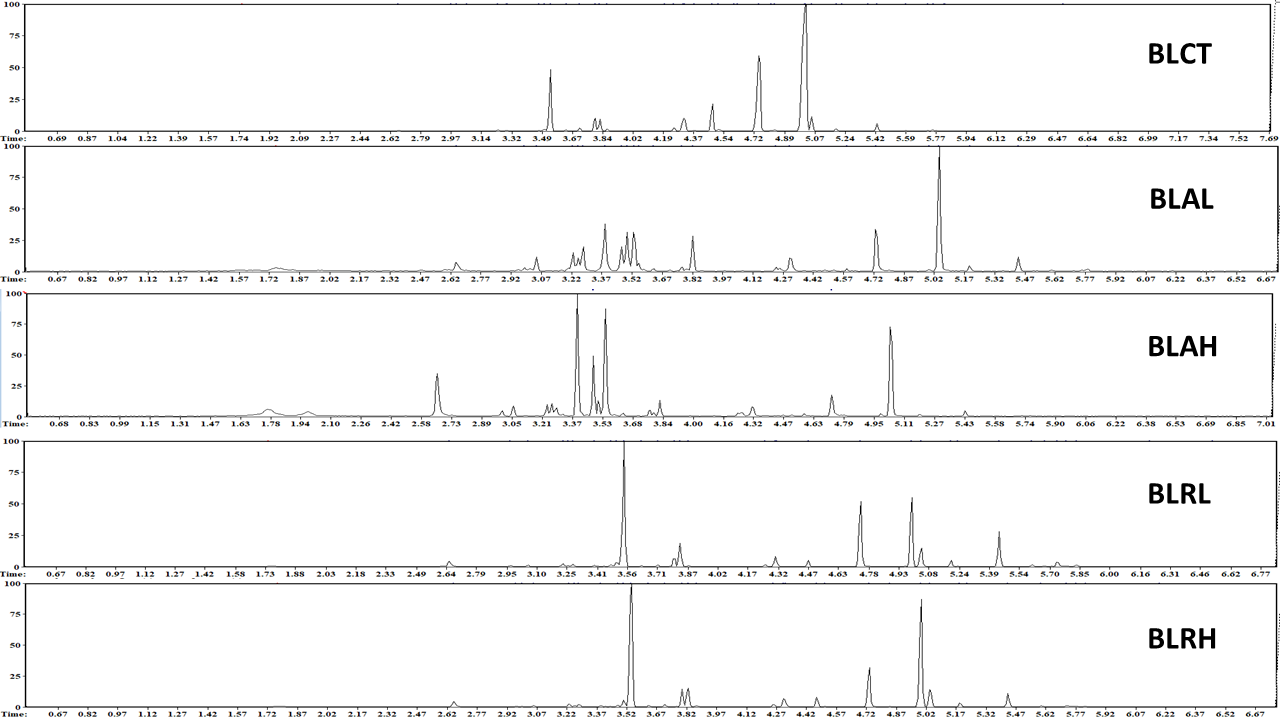


**Figure S1**: Representative GC-MS chromatogram of analyzed bay leaf samples before and after exposure to autoclaving and γ-radiation low and high doses (samples codes abbreviations were listed in table 1).


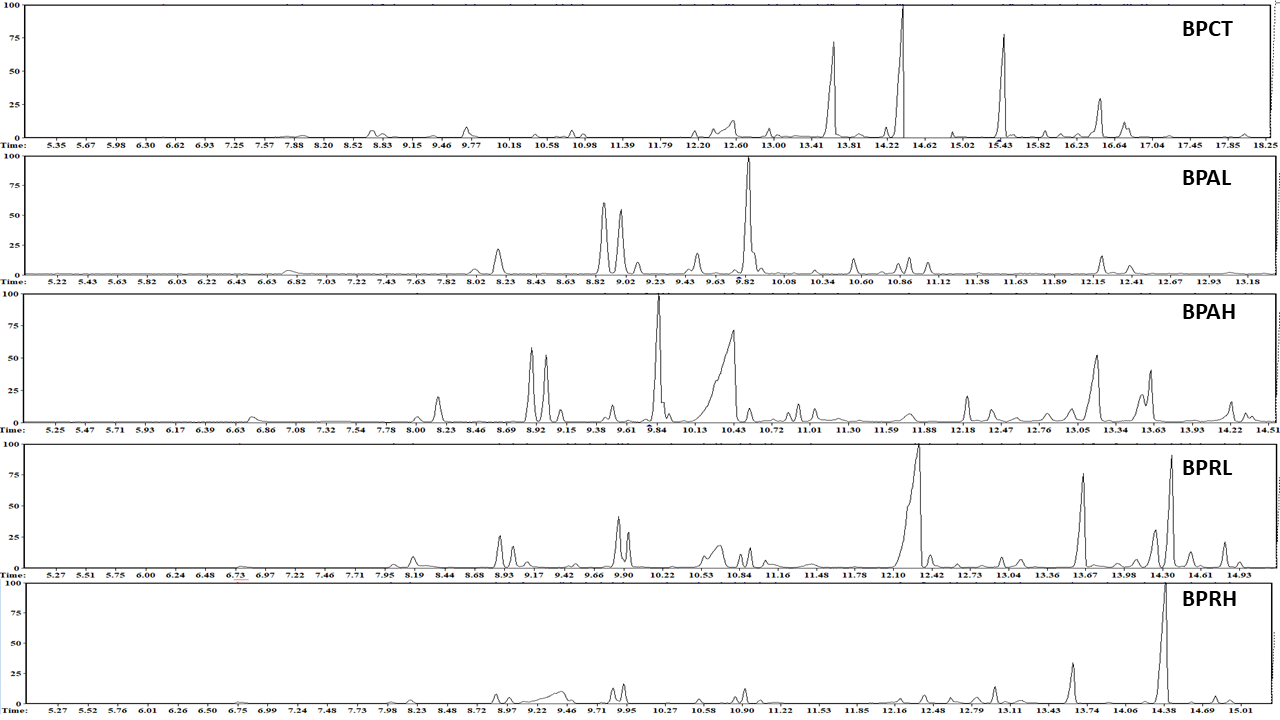


**Figure S2:** Representative GC-MS chromatogram of analyzed black pepper samples before and after exposure to autoclaving and γ-radiation low and high doses.


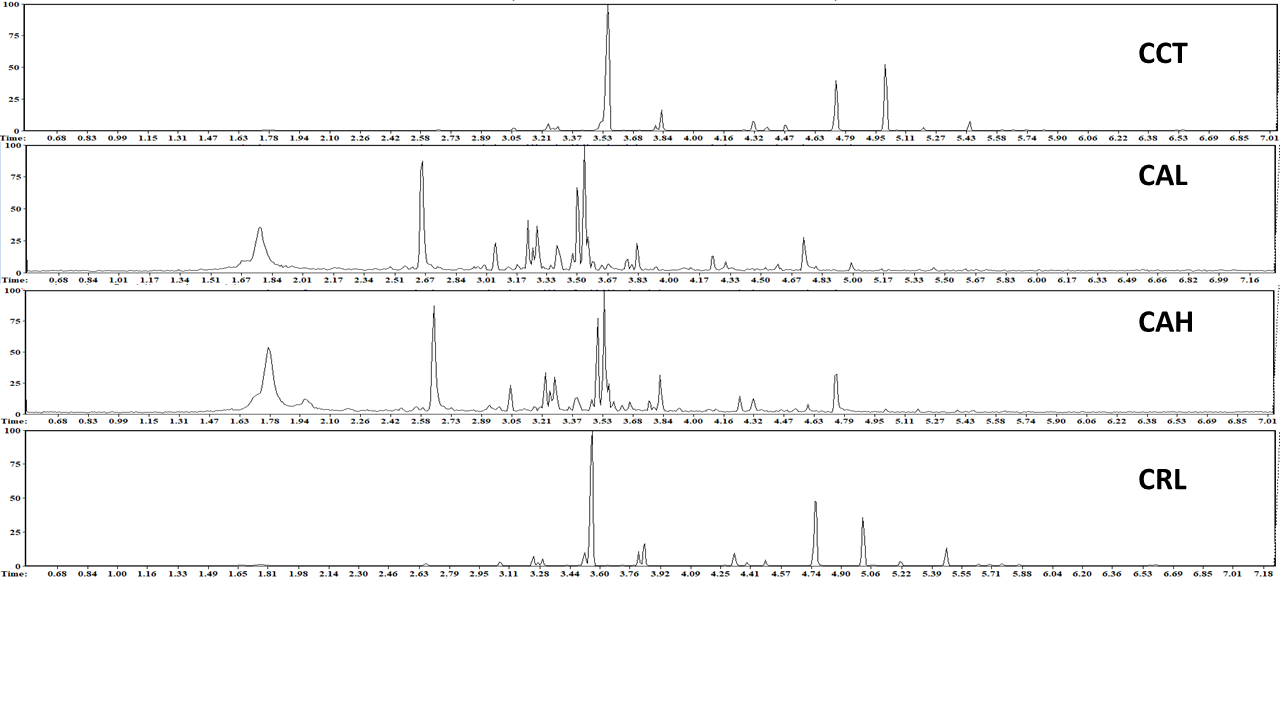


**Figure S3:** Representative GC-MS chromatogram of analyzed capsicum samples before and after exposure to autoclaving and γ-radiation low and high doses.


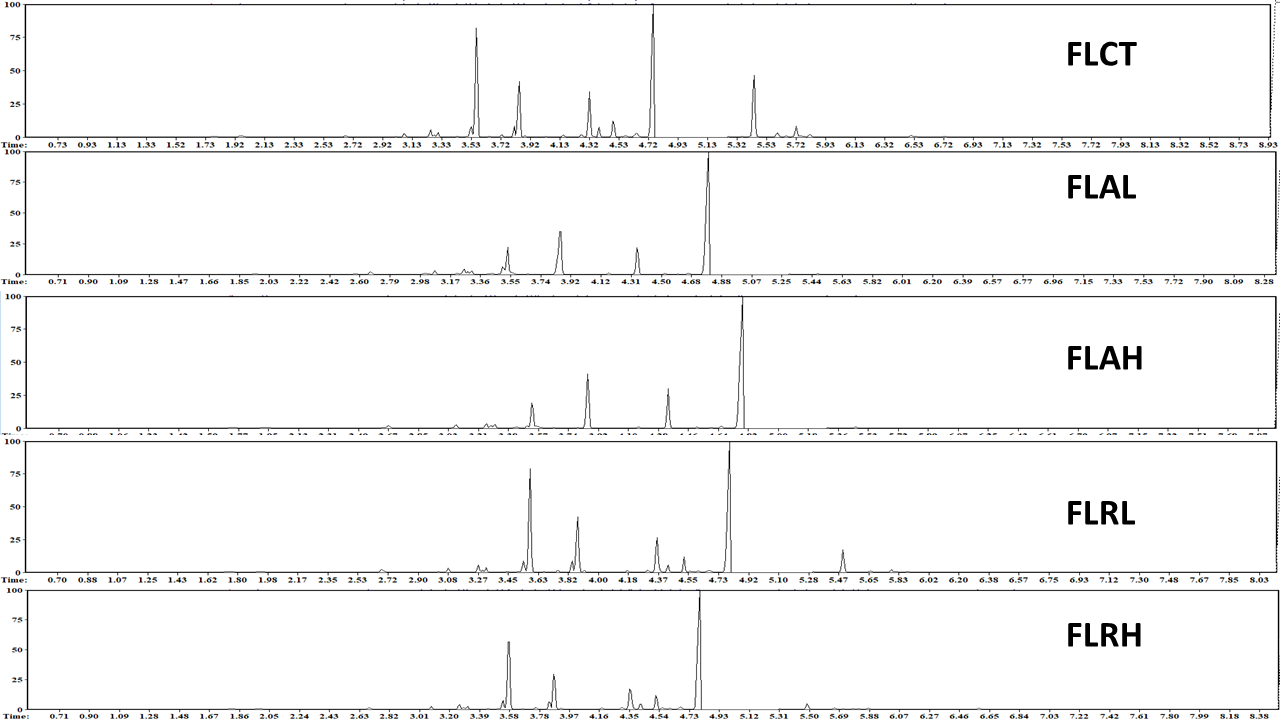


**Figure S4:** Representative GC-MS chromatogram of analyzed fennel samples before and after exposure to autoclaving and γ-radiation low and high doses.


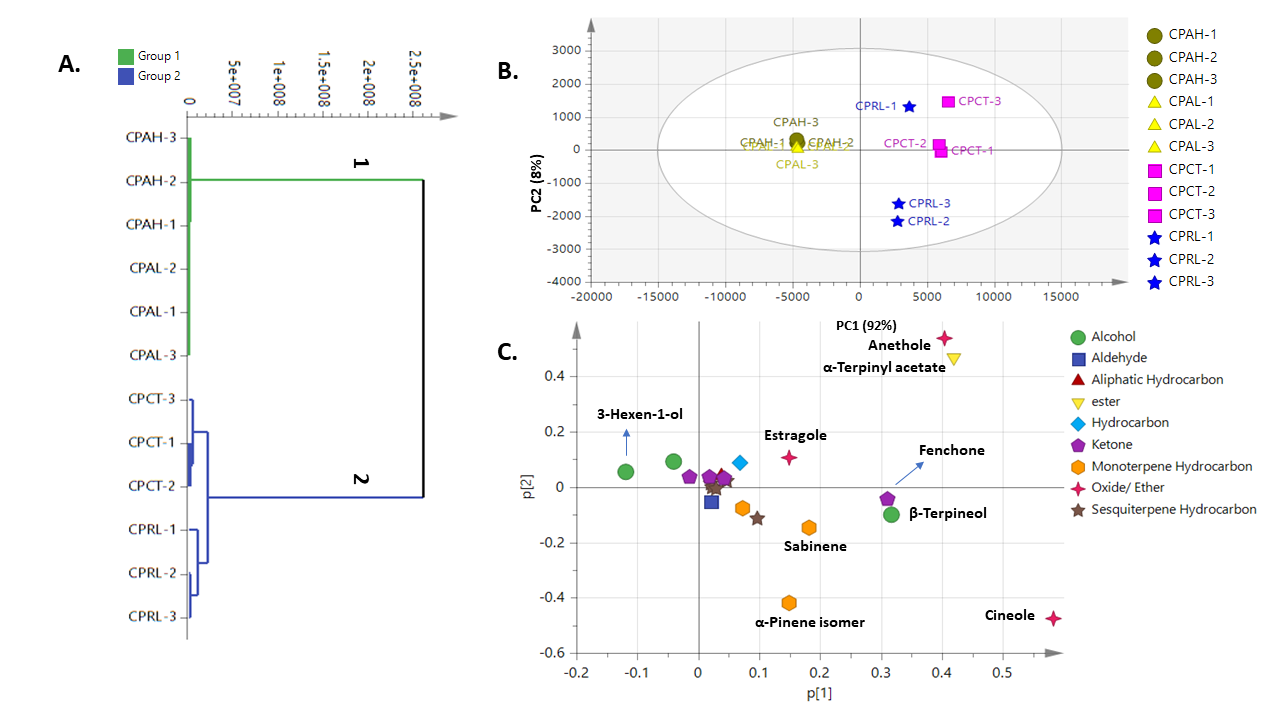


**Figure S5.** PCA of Capsicum samples’ in control and post treatment clusters as described by two vectors of the principal component PC1 (92%) and PC2 (8%). (A) HCA dendrogram for different Capsicum samples. (B) Score plot of PC1 vs. PC2. (C) Loading plot revealing metabolite variants between clusters.


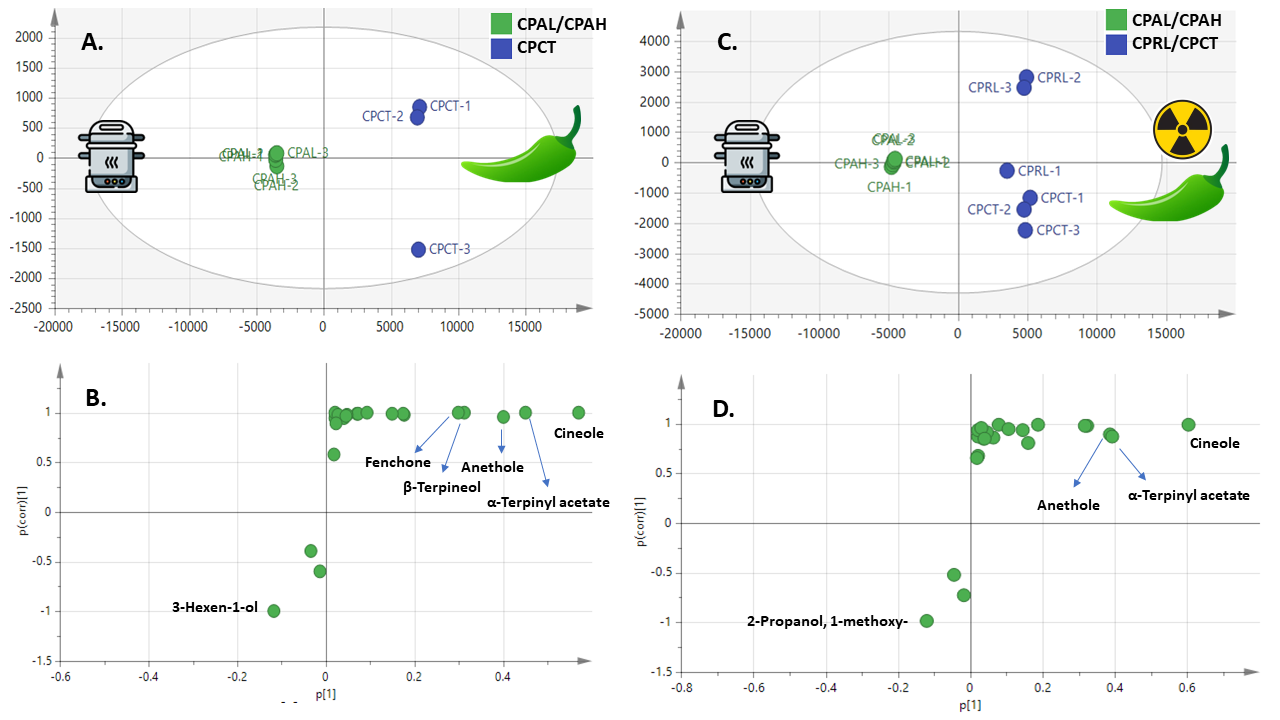


**Figure S6.** OPLS-DA model for Capsicum treated versus control samples. (A) Score plot based on GCMS data for control versus autoclaved samples. (B) S-plot for Capsicum control and autoclaved samples showing covariance p [1] against the correlation p(cor) [1] for the variables of discriminating components, (p < 0.05). (C) Score plot based on GCMS data for control and irradiated samples versus autoclaved samples. (D) S-plot for Capsicum control irradiated and autoclaved samples showing covariance p [1] against the correlation p(cor) [1] for the variables of discriminating components, (p < 0.05).


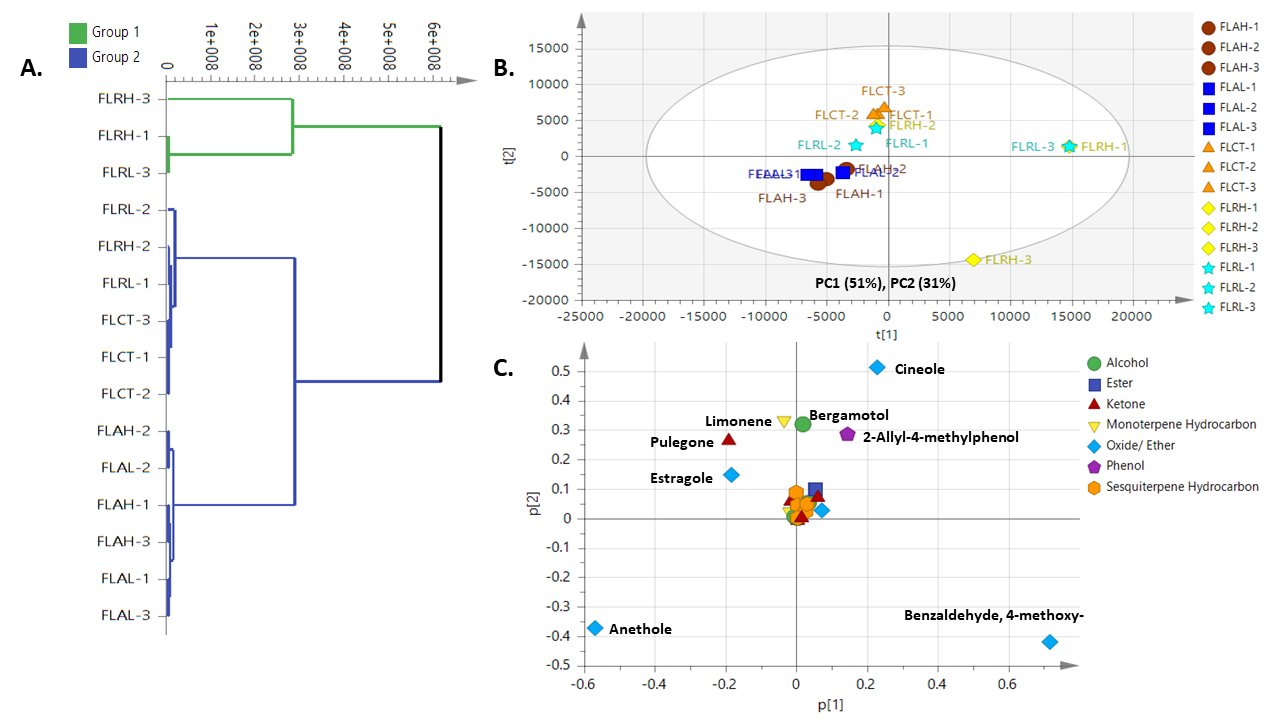


**Figure S7.** PCA of Fennel samples’ in control and post treatment clusters as described by two vectors of the principal component PC1 (51%) and PC2 (31%). (A) HCA dendrogram for different Fennel samples. (B) Score plot of PC1 vs. PC2. (C) Loading plot revealing metabolite variants between clusters**.**


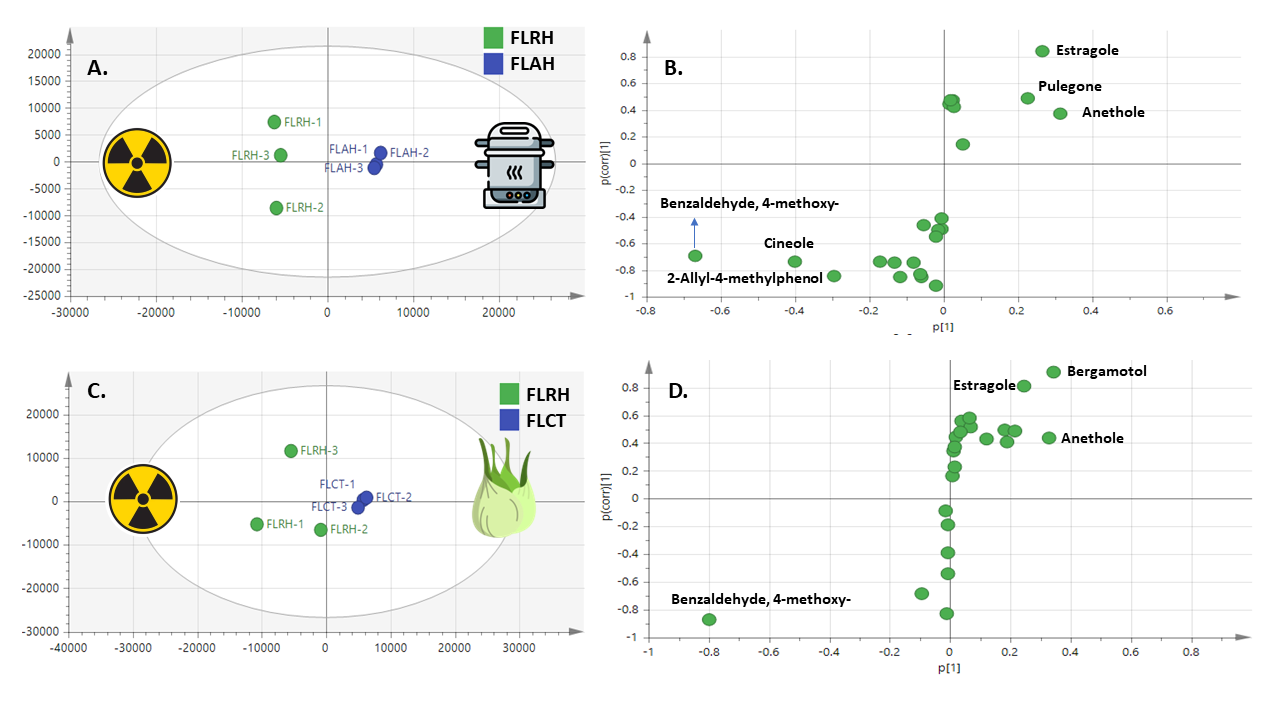


**Figure S8.** OPLS-DA model for Fennel treated versus control samples. (A) Score plot based on GCMS data for autoclaved high versus irradiated high samples. (B) S-plot for irradiated and autoclaved Fennel samples showing covariance p [1] against the correlation p(cor) [1] for the variables of discriminating components, (p > 0.05). (C) Score plot based on GCMS data for control versus irradiated high samples. (D) S-plot for Fennel control and irradiated samples showing covariance p [1] against the correlation p(cor) [1] for the variables of discriminating components, (p > 0.05).

| **Table S1.** The chemical composition of black pepper essential oils recovered from un-treated leaves (BPCT), low-dose autoclave (BPAL), high-dose autoclave (BPAH), low-dose γ-radiated (BPRL), and high-dose γ-radiated (BPRH). | | | | | | | | | |
| --- | --- | --- | --- | --- | --- | --- | --- | --- | --- |
| Peak No | **Average Rt(min)** | **Average RI** | **Metabolite name** | **Chemical Class** | **BPCT (% ± SE)** | **BPAL (% ± SE)** | **BPAH (% ± SE)** | **BPRL (% ± SE)** | **BPRH (% ± SE)** |
| 1 | 7.08 | 915 | 3-Hexenol | Alcohol | Traces | 0.01 ±0.001 | 0.01 ±0.001 | Traces | Traces |
| 14 | 10.55 | 1148 | 4-Thujanol | Alcohol | 1.89 ±0.67 | 2.92 ±0.09 | 1.71 ±0.18 | 1.65 ±0.10 | 1.33 ±0.10 |
| 16 | 10.84 | 1170 | Linalool | Alcohol | 0.71 ±0.32 | 2.21 ±0.18 | 1.24 ±0.15 | 6.59 ±3.65 | 2.04 ±0.06 |
| 18 | 11.01 | 1182 | β-Terpineol | Alcohol | Traces | 0.60 ±0.57 | 0.02 ±0.001 | 0.05 ±0.03 | 0.02 ±0.01 |
| 20 | 12.20 | 1278 | 4-Terpinenol | Alcohol | 0.78 ±0.62 | 4.06 ±0.52 | 3.27 ±0.30 | 1.84 ±0.21 | 1.27 ±0.05 |
| **Total Alcohol** | | | | | **3.39** | **9.81** | **6.24** | **10.14** | **4.66** |
| 28 | 14.35 | 1470 | α-Terpinyl acetate | Ester | 25.94 ±1.98 | Traces | 1.19 ±0.40 | 5.39 ±5.37 | 22.86 ±11.43 |
| **Total Ester** | | | | | **25.94** | **0.00** | **1.19** | **5.39** | **22.86** |
| 17 | 10.98 | 1180 | Fenchone | Ketone | Traces | 0.05 ±0.03 | 0.01 ±0.01 | 0.01 ±0.001 | 0.01 ±0.01 |
| **Total Ketone** | | | | | **0.00** | **0.05** | **0.01** | **0.01** | **0.01** |
| 2 | 7.48 | 939 | α-Thujene | Monoterpene hydrocarbon | Traces | 0.02 ±0.001 | 0.01 ±0.001 | Traces | Traces |
| 3 | 7.90 | 965 | α-Pinene | Monoterpene hydrocarbon | Traces | 0.01 ±0.001 | 0.01 ±0.001 | Traces | 0.01 ±0.001 |
| 3 | 8.17 | 982 | β-Thujene | Monoterpene hydrocarbon | 0.34 ±0.32 | 9.08 ±0.54 | 5.70 ±0.59 | 4.54 ±0.19 | 2.28 ±0.09 |
| 4 | 8.87 | 1028 | β-Thujene isomer | Monoterpene hydrocarbon | 1.10 ±0.90 | 22.49 ±1.14 | 17.13 ±0.80 | 9.39 ±0.43 | 5.25 ±0.17 |
| 5 | 8.98 | 1035 | Sabinen | Monoterpene hydrocarbon | 3.75 ±1.27 | 19 ±0.34 | 13.52 ±0.62 | 6.31 ±0.18 | 3.21 ±0.15 |
| 6 | 9.04 | 1039 | β-Pinene | Monoterpene hydrocarbon | Traces | 0.02 ±0.01 | 0.01 ±0.001 | 0.01 ±0.001 | 0.04 ±0.04 |
| 7 | 9.10 | 1044 | β-Myrcene | Monoterpene hydrocarbon | 1.50 ±0.70 | 4.96 ±0.26 | 11.05 ±6.99 | 2.24 ±0.22 | 1.42 ±0.11 |
| 8 | 9.49 | 1070 | α-Phellandrene | Monoterpene hydrocarbon | 0.62 ±0.07 | 3.41 ±0.18 | 2.68 ±0.06 | 1.48 ±0.21 | 3.82 ±2.98 |
| 9 | 9.66 | 1082 | Isoterpinolene | Monoterpene hydrocarbon | Traces | 0.17 ±0.14 | 0.01 ±0.001 | 0.02 ±0.02 | 0.06 ±0.06 |
| 10 | 9.75 | 1088 | Limonene | Monoterpene hydrocarbon | 0.26 ±0.08 | 1.69 ±0.09 | 1.06 ±0.19 | 0.89 ±0.08 | 0.59 ±0.04 |
| 11 | 9.80 | 1092 | β-Ocimene | Monoterpene hydrocarbon | 0.01 ±0.01 | 0.04 ±0.01 | 0.03 ±0.02 | 0.04 ±0.01 | 0.03 ±0.01 |
| 12 | 9.84 | 1094 | m-Cymene | Monoterpene hydrocarbon | 3.84 ±0.53 | 21.77 ±1.12 | 14.73 ±2.05 | 10.06 ±0.54 | 5.16 ±0.44 |
| **Total Monoterpene hydrocarbon** | | | | | **11.44** | **82.67** | **65.93** | **34.97** | **21.88** |
| 13 | 9.93 | 1101 | Cineole | Oxide/ Phenol/ Ether | 1.56 ±0.61 | 2.08 ±0.30 | 1.49 ±0.17 | 8.58 ±0.85 | 7.13 ±0.98 |
| 21 | 12.40 | 1295 | Anethole | Oxide/ Phenol/ Ether | 1.17 ±0.24 | 2.69 ±0.91 | 0.93 ±0.08 | 1.97 ±0.23 | 1.39 ±0.03 |
| 26 | 13.62 | 1402.52 | Estragole | Oxide/ Phenol/ Ether | 25.91 ±0.73 | 0.06 ±0.06 | 7.32 ±0.51 | 29.44 ±5.54 | 30.43 ±13.43 |
|  |  |  | **Total Oxide/ Ether** |  | **28.64** | **0.00** | **9.75** | **39.99** | **38.95** |
| 15 | 10.72 | 1161 | Caryophyllene | Sesquiterpene hydrocarbon | 0.01 ±0.001 | 0.07 ±0.04 | 0.01 ±0.001 | 0.18 ±0.11 | 0.02 ±0.02 |
| 19 | 11.75 | 1241 | Humulene | Sesquiterpene hydrocarbon | 0.38 ±0.24 | 0.26 ±0.02 | 5.59 ±4.58 | 0.19 ±0.05 | 4.03 ±1.99 |
| 22 | 12.98 | 1346 | Germacrene D | Sesquiterpene hydrocarbon | 2.42 ±0.28 | 1.06 ±0.82 | 0.68 ±0.22 | 2.62 ±0.32 | 5.37 ±0.19 |
| 23 | 13.09 | 1356 | β-Farnesene | Sesquiterpene hydrocarbon | 0.77 ±0.16 | 0.35 ±0.17 | 4.09 ±3.90 | 0.30 ±0.06 | 0.89 ±0.19 |
| 24 | 13.12 | 1358 | α-Muurolene | Sesquiterpene hydrocarbon | 0.13 ±0.11 | 0.05 ±0.04 | 1.43 ±0.72 | 0.24 ±0.19 | 0.13 ±0.12 |
| 25 | 13.39 | 1381 | δ-Cadinene | Sesquiterpene hydrocarbon | 0.20 ±0.10 | 0.85 ±0.75 | 2.30 ±1.25 | 4.33 ±4.29 | 0.71 ±0.18 |
| 27 | 14.23 | 1459 | δ-EIemene | Sesquiterpene hydrocarbon | 1.50 ±0.42 | Traces | 2.20 ±0.11 | 0.67 ±0.64 | 0.46 ±0.17 |
| 29 | 14.46 | 1481 | α-Cubebene | Sesquiterpene hydrocarbon | 0.25 ±0.04 | Traces | 0.42 ±0.01 | 0.97 ±0.97 | 0.01 ±0.01 |
| 30 | 15.42 | 1565 | Caryophyllene | Sesquiterpene hydrocarbon | 10.33 ±1.03 | Traces | 0.15 ±0.15 | Traces | 0.01 ±0.01 |
| 31 | 15.98 | 1610 | Humulene | Sesquiterpene hydrocarbon | 1.60 ±0.23 | Traces | Traces | Traces | 0.01 ±0.001 |
| 32 | 16.55 | 1649 | α-Bisabolene | Sesquiterpene hydrocarbon | 10.09 ±2.01 | Traces | Traces | Traces | Traces |
| 33 | 16.82 | 1667 | Copaene | Sesquiterpene hydrocarbon | 2.90 ±0.74 | Traces | Traces | Traces | Traces |
| **Total Sesquiterpene hydrocarbon** | | | | | **30.58** | **2.65** | **16.88** | **9.50** | **11.64** |

| **Table S2.** Major metabolites detected in different spices. Statistical analysis was carried out using one-way ANOVA where unshared letters between groups are significance at *p* ≤ 0.05. | | | | | |
| --- | --- | --- | --- | --- | --- |
| Capsicum | | | | | |
|  | "Acetic acid" | 3-Hexen-1-ol | Sabinene | ß-Terpineol | Cineole |
| CT vs RH | - | - | - | - | - |
| CT vs RL | ns | ns | ns | ns | ns |
| CT vs AH | *** | *** | ns | *** | *** |
| CT vs AL | *** | *** | * | *** | *** |
| Bay leaf | | | | | |
|  | 4-Hexen-1-ol | Cineole | Sabinene | Estragole | Fenchone |
| CT vs RH | ns | *** | ns | ns | * |
| CT vs RL | ns | ** | ns | ns | ** |
| CT vs AH | *** | *** | ns | *** | ns |
| CT vs AL | *** | ns | * | * | *** |
| Fennel | | | | | |
|  | limonene | Cineole | Pulegone | Estragole | 2-Allyl-4-methylphenol |
| CT vs RH | ns | ns | ns | ns | ns |
| CT vs RL | ns | ns | ns | ns | ns |
| CT vs AH | ns | ** | ns | ns | ** |
| CT vs AL | ns | ** | ns | ns | ** |
| Black pepper | | | | | |
|  | ß-Thujene | Sabinene | m-Cymene | Cineole | E-Anethole |
| CT vs RH | ns | ns | ns | ns | ns |
| CT vs RL | ns | ns | ns | ns | ns |
| CT vs AH | ** | ** | * | ns | ns |
| CT vs AL | ** | *** | ** | ns | * |
| ns: p< 0.05, *: p≤0.05, **: p$\leq$0.01, ***: p≤0.001 | | | | | |
